# Impact of pre-existing cardiovascular disease on treatment patterns and survival outcomes in patients with lung cancer

**DOI:** 10.1186/s12885-020-07487-9

**Published:** 2020-10-15

**Authors:** Atul Batra, Dropen Sheka, Shiying Kong, Winson Y. Cheung

**Affiliations:** 1 Department of Medical Oncology, Tom Baker Cancer Center, 1331 29 ST NW, Calgary, Alberta T2N 4N2 Canada; 2 University of Calgary, Calgary, Alberta Canada

**Keywords:** Lung cancer, Cardiovascular disease, Cardio-oncology, Treatment trends, Survival outcomes

## Abstract

**Background:**

Baseline cardiovascular disease (CVD) can impact the patterns of treatment and hence the outcomes of patients with lung cancer. This study aimed to characterize treatment trends and survival outcomes of patients with pre-existing CVD prior to their diagnosis of lung cancer.

**Methods:**

We conducted a retrospective, population-based cohort study of patients with lung cancer diagnosed from 2004 to 2015 in a large Canadian province. Multivariable logistic regression and Cox regression models were constructed to determine the associations between CVD and treatment patterns, and its impact on overall (OS) and cancer-specific survival (CSS), respectively. A competing risk multistate model was developed to determine the excess mortality risk of patients with pre-existing CVD.

**Results:**

A total of 20,689 patients with lung cancer were eligible for the current analysis. Men comprised 55%, and the median age at diagnosis was 70 years. One-third had at least one CVD, with the most common being congestive heart failure in 15% of patients. Pre-existing CVD was associated with a lower likelihood of receiving chemotherapy (odds ratio [OR], 0.53; 95% confidence interval [CI], 0.48–0.58; *P* < .0001), radiotherapy (OR, 0.76; 95% CI, 0.7–0.82; *P* < .0001), and surgery (OR, 0.56; 95% CI, 0.44–0.7; *P* < .0001). Adjusting for measured confounders, the presence of pre-existing CVD predicted for inferior OS (hazard ratio [HR], 1.1; 95% CI, 1.1–1.2; *P* < .0001) and CSS (HR, 1.1; 95% CI, 1.1–1.1; *P* < .0001). However, in the competing risk multistate model that adjusted for baseline characteristics, prior CVD was associated with increased risk of non-cancer related death (HR, 1.48; 95% CI, 1.33–1.64; *P* < 0.0001) but not cancer related death (HR, 0.98; 95% CI, 0.94–1.03; *P* = 0.460).

**Conclusions:**

Patients with lung cancer and pre-existing CVD are less likely to receive any modality of cancer treatment and are at a higher risk of non-cancer related deaths. As effective therapies such as immuno-oncology drugs are introduced, early cardio-oncology consultation may optimize management of lung cancer.

## Background

Cardiovascular disease and cancer are the two leading causes of death worldwide, and account for approximately 17.9 million and 9.6 million deaths annually across the globe, respectively [1–3]. The relationship between these two comorbid conditions is complex. While cardiovascular disease and cancer share common modifiable risk factors and pathophysiological mechanisms, the treatments and outcomes of one may be affected by the other [4–9]. Further, cancer treatments, including chemotherapy, targeted therapy, and radiotherapy, have been associated with increased downstream cardiovascular sequelae, independent of other risk factors [10].

For example, cigarette smoking is estimated to contribute to 71% of lung cancer related mortality and 10% of deaths due to cardiovascular disease, respectively [11]. Inflammation and oxidative stress are key mechanisms in the pathophysiology of both conditions [12–14]. The other link between lung cancer and cardiovascular disease is advanced age. The median age at the diagnosis of cardiovascular disease is 65 years and that for lung cancer is 70 years [15, 16].

Previous studies have primarily focused on the long-term cardiovascular complications of cancer treatments [17, 18]. Data related to the prevalence of pre-existing cardiovascular diseases in patients diagnosed with cancer has been reported to range from 5 to 43%, depending on the primary site of malignancy and the geographical region where the study was conducted [19, 20]. However, there are limited studies reporting on the impact of pre-existing cardiovascular disease on subsequent treatment patterns and survival outcomes of lung cancer. A large study of patients with breast cancer concluded that those with baseline cardiovascular disease were less likely to receive any form of therapy (surgery, chemotherapy, hormonal therapy, and radiotherapy) compared to patients without pre-existing cardiovascular comorbidities [21]. Further, overall survival but not cancer specific survival was shorter in these patients. While a few studies have described inferior survival outcomes among patients with lung cancer and pre-existing cardiovascular disease, the effect of treatments on prognosis remains largely unknown [22, 23].

In this study, we aimed to identify the proportion of patients who had at least one pre-existing cardiovascular disease at the time of their diagnosis of lung cancer. Further, we analyzed the effect of pre-existing cardiovascular disease on treatment patterns, including chemotherapy, radiation therapy, and surgery, as well as its impact on survival outcomes in patients with lung cancer.

## Methods

### Data sources and study population

The study was conducted in Alberta, which is a large province in Canada, with a population of over 4 million people and has a single-payer, universal healthcare system. This was a retrospective, population-based study, where we compiled data from various administrative sources including the Alberta cancer registry (ACR), hospital discharge abstracts, national ambulatory care reporting system, provincial physician billing claims, vital statistics, and the 2011 census.

The ACR prospectively records data on all residents diagnosed with cancer in the province, including demographic variables, tumor characteristics, and primary treatment patterns. The discharge abstracts provide data collected during hospital admissions and details up to 25 International Classification of Diseases tenth version (ICD-10) diagnoses and up to 20 procedures coded as per the Canadian Classification of Health Interventions. Likewise, the ambulatory care data record up to 10 diagnoses and 10 procedures, respectively. The provincial physician billing claims data provide the location of healthcare delivery, physician specialty, one procedure code, and up to three ICD-10 diagnoses. Each patient in the province has a unique provincial health number, which was used to link the data from the various sources. Data on death from lung cancer or other causes were retrieved from vital statistics.

Patients aged 18 years and older with a diagnosis of lung cancer between January 1, 2004, and December 31, 2015, in Alberta, Canada were included in the current analysis. Patients who moved out of Alberta within 12 months of primary treatment, those who had multiple primary tumors, or who did not have an Alberta healthcare number were excluded.

Approval was obtained from the Health Research Ethics Board of Alberta’s Cancer Committee prior to commencing this study.

### Clinical variables and outcomes

#### Outcomes

The main outcomes of this study were treatment patterns and survival outcomes. We assessed the impact of pre-existing cardiovascular disease on treatments, including the receipt of any chemotherapy, surgery, and radiotherapy. Primary surgery type was identified as the most definitive surgical procedure (lobectomy or segmental resection or pneumonectomy) performed within 1 year of diagnosis. Information on the administration of chemotherapy and radiotherapy was categorized in a binary fashion (yes or no), which was obtained via the ACR. Survival outcomes including overall survival (OS) and cancer specific survival (CSS) were retrieved from vital statistics records.

#### Independent variables

Using data from the above sources, the presence of pre-existing cardiovascular disease at or preceding the time of diagnosis of lung cancer was identified using previously validated ICD algorithms [24]. Cardiovascular diseases included the presence of congestive heart failure, myocardial infarction, cerebrovascular accident and/or arrhythmias. Tumor characteristics included the American Joint Committee on Cancer stage and histologic type or subtype (adenocarcinoma, squamous cell carcinoma, small cell lung cancer, carcinoid, large cell cancer, and others). The discharge abstracts, physician billing claims, and ambulatory care data were used to derive the Charlson comorbidity index. Alberta is strategically divided into five health zones for healthcare delivery. Postal code at diagnosis was used to assign each patient to their respective health zone (Calgary, Edmonton, North, Central, or South) and to retrieve neighbourhood-level socioeconomic status (education and income levels) based on the 2011 census. Driving time to the nearest oncology facility was calculated using Google Maps interface and postal codes, and it was further categorized as one, one to two and more than 2 h, as per previously published literature [25].

### Statistical analysis

Baseline characteristics were summarized using descriptive statistics, where the Student’s t test and Wilcoxon rank-sum test were used to compare continuous variables, and the chi-square tests were applied to compare categorical variables across the different patient groups. Multivariable logistic regression analyses were performed to identify associations between pre-existing cardiovascular disease and receipt of treatment (including chemotherapy, radiotherapy, and surgery). We also performed age and histology stratified logistic regression analyses for each treatment modality. Kaplan-Meier curves were plotted to estimate OS and CSS differences between patients with and without cardiovascular disease, and compared using log rank tests. Cox proportional hazards models were constructed to examine the effect of pre-existing cardiovascular disease on OS and CSS, respectively. The proportional hazard assumptions for the regression models were tested using Schoenfeld residuals. These models were further stratified by age and histology. OS and CSS were defined as the time interval between the date of cancer diagnosis and the date of death from any cause, and the date of death from lung cancer, respectively. Further, we also constructed a competing risk multistate model with three states: alive, cancer related death and non-cancer related death, to examine the excess mortality risk of patients with pre-existing cardiovascular disease. Subgroup analyses were performed to investigate whether the effects of cardiovascular disease on outcomes were modified by different treatments, including chemotherapy, surgery, and radiotherapy. All tests were two-sided, and the significance level was defined a priori as 0.05. All analyses were performed with SAS statistical software version 9.4 (SAS Institute, Inc., Cary, NC).

## Results

### Patient characteristics

A total of 20,689 patients were diagnosed with lung cancer during the study period. The median age at diagnosis was 70 (interquartile range, 62–78) years, and women constituted 49% of the cohort. Approximately half of patients had distant metastases at diagnosis. Adenocarcinoma (37.3%) and squamous cell carcinoma (17.8%) were the most common histologies, while small cell lung cancer was diagnosed in 12.4%. Surgery was performed in 16.5%, and chemotherapy and radiation therapy were administered in 27.4 and 30.3% of patients, respectively. Only 20 patients (0.1%) received immunotherapy. Additional baseline characteristics are shown in Table 1.

**Table 1:** Baseline characteristics of patients with lung cancer

Variables	Total (***N*** = 20,689), n (%)	With cardiovascular disease (***N*** = 6436), n (%)	Without cardiovascular disease (***N*** = 14,253), n (%)	***P***-value
**Age, in years**				< 0.0001
Mean (+STD)	69.5 (±11)	73.7 (±9.7)	67.6 (±11.1)	
Median (IQR)	70 (62–78)	75 (67–81)	68 (60–76)	
**Age group**				< 0.0001
< =60	4443 (21.5%)	658 (10.2%)	3785 (26.6%)	
61–70	6112 (29.5%)	1549 (24.1%)	4563 (32%)	
71–80	6717 (32.5%)	2590 (40.2%)	4127 (29%)	
> 80	3417 (16.5%)	1639 (25.5%)	1778 (12.5%)	
**Sex**				< 0.0001
Female	10,156 (49.1%)	2874 (44.7%)	7282 (51.1%)	
Male	10,533 (50.9%)	3562 (55.3%)	6971 (48.9%)	
**Year of diagnosis**				0.0337
2004–2009	9993 (48.3%)	3038 (47.2%)	6955 (48.8%)	
2010–2015	10,696 (51.7%)	3398 (52.8%)	7298 (51.2%)	
**CCI score**				< 0.0001
0	2566 (12.4%)	310 (4.8%)	2256 (15.8%)	
1	2614 (12.6%)	627 (9.7%)	1987 (13.9%)	
> 1	15,509 (75%)	5499 (85.4%)	10,010 (70.2%)	
**Histology**				< 0.0001
Adenocarcinoma	7727 (37.3%)	2024 (31.4%)	5703 (40%)	
SCC	3680 (17.8%)	1184 (18.4%)	2496 (17.5%)	
SCLC	2571 (12.4%)	799 (12.4%)	1772 (12.4%)	
Carcinoid/LCNE	520 (2.5%)	117 (1.8%)	403 (2.8%)	
Large cell cancer	260 (1.3%)	59 (0.9%)	201 (1.4%)	
Others	5931 (28.7%)	2253 (35%)	3678 (25.8%)	
**Stage**				
I	3636 (17.6%)	1127 (17.5%)	2509 (17.6%)	< 0.0001
II	880 (4.3%)	250 (3.9%)	630 (4.4%)	
III	5168 (25%)	1591 (24.7%)	3577 (25.1%)	
IV	10,409 (50.3%)	3207 (49.8%)	7202 (50.5%)	
Unknown	596 (2.9%)	261 (4.1%)	335 (2.4%)	
**T stage**				
T0	110 (0.5%)	44 (0.7%)	66 (0.5%)	< 0.0001
T1	3095 (15%)	985 (15.3%)	2110 (14.8%)	
T2	6581 (31.8%)	2001 (31.1%)	4580 (32.1%)	
T3	788 (3.8%)	188 (2.9%)	600 (4.2%)	
T4	9378 (45.3%)	2966 (46.1%)	6412 (45%)	
Unknown	737 (3.6%)	252 (3.9%)	485 (3.4%)	
**N stage**				
N0	6540 (31.6%)	2126 (33%)	4414 (31%)	< 0.0001
N1	1729 (8.4%)	494 (7.7%)	1235 (8.7%)	
N2	7453 (36%)	2302 (35.8%)	5151 (36.1%)	
N3	3803 (18.4%)	1073 (16.7%)	2730 (19.2%)	
Unknown	1164 (5.6%)	441 (6.9%)	723 (5.1%)	
**M stage**				
M0	9877 (47.7%)	3069 (47.7%)	6808 (47.8%)	0.0008
M1	10,409 (50.3%)	3207 (49.8%)	7202 (50.5%)	
Unknown	403 (1.9%)	160 (2.5%)	243 (1.7%)	
**Laterality**				
Bilateral	211 (1%)	74 (1.1%)	137 (1%)	0.0003
Left	8477 (41%)	2677 (41.6%)	5800 (40.7%)	
Right	11,568 (55.9%)	3515 (54.6%)	8053 (56.5%)	
Unknown	433 (2.1%)	170 (2.6%)	263 (1.8%)	
**Surgery**				
No	17,274 (83.5%)	5721 (88.9%)	11,553 (81.1%)	< 0.0001
Lobectomy or segmental	3168 (15.3%)	686 (10.7%)	2482 (17.4%)	
Pneumonectomy	247 (1.2%)	29 (0.5%)	218 (1.5%)	
**Chemotherapy**				
No	15,029 (72.6%)	5378 (83.6%)	9651 (67.7%)	< 0.0001
Yes	5660 (27.4%)	1058 (16.4%)	4602 (32.3%)	
**Immunotherapy**				
No	20,669 (99.9%)	6433 (100%)	14,236 (99.9%)	0.1195
Yes	20 (0.1%)	3 (0%)	17 (0.1%)	
**Radiation**				
No	14,418 (69.7%)	4743 (73.7%)	9675 (67.9%)	< 0.0001
Yes	6271 (30.3%)	1693 (26.3%)	4578 (32.1%)	
**Institution type**				
Academic	10,713 (51.8%)	3119 (48.5%)	7594 (53.3%)	< 0.0001
Community	9976 (48.2%)	3317 (51.5%)	6659 (46.7%)	
**Driving time (hours)**				
1	15,419 (74.5%)	4704 (73.1%)	10,715 (75.2%)	< 0.0001
2	2885 (13.9%)	1010 (15.7%)	1875 (13.2%)	
> 2	2385 (11.5%)	722 (11.2%)	1663 (11.7%)	
**Health Zone**				
Calgary	6192 (29.9%)	1781 (27.7%)	4411 (30.9%)	< 0.0001
Central	3125 (15.1%)	1045 (16.2%)	2080 (14.6%)	
Edmonton	7159 (34.6%)	2239 (34.8%)	4920 (34.5%)	
North	2459 (11.9%)	755 (11.7%)	1704 (12%)	
South	1754 (8.5%)	616 (9.6%)	1138 (8%)	
**Educational level**^a^				
< = 80%	10,133 (49%)	3281 (51%)	6852 (48.1%)	0.0005
> 80%	10,527 (50.9%)	3147 (48.9%)	7380 (51.8%)	
Unknown	29 (0.1%)	8 (0.1%)	21 (0.1%)	
**Income level**				
< = 46 k	12,849 (62.1%)	4085 (63.5%)	8764 (61.5%)	0.0237
> 46 k	7811 (37.8%)	2343 (36.4%)	5468 (38.4%)	
Unknown	29 (0.1%)	8 (0.1%)	21 (0.1%)	

*STD* Standard deviation, *IQR* Interquartile range, *CCI* Charlson’s comorbidity index, *SCC* Squamous cell cancer, *SCLC* Small cell lung cancer, *LCNE* Large Cell Neuroendocrine tumor. ^a^80% of residents have high school and above level of education in the neighborhood

### Pre-existing cardiovascular diseases

Of all patients with lung cancer, 6436 (31.1%) had at least one pre-existing cardiovascular disease. The most common pre-existing cardiovascular disease at the diagnosis of lung cancer included congestive heart failure (47.4%), followed by previous cerebrovascular accident (40.3%), myocardial infarction (33.0%), and chronic arrythmias (30.4%), respectively (Table 2).

**Table 2:** Type of cardiovascular disease

Cardiovascular disease	Number (percentage of those with cardiovascular disease) (***n*** = 6436)	Percentage of overall population (***n*** = 20,689)
Previous myocardial infarction	2127 (33%)	10.3%
Congestive heart failure	3049 (47.4%)	14.7%
Cerebrovascular accident	2596 (40.3%)	12.5%
Chronic arrhythmias	1954 (30.4%)	9.4%

Patients with pre-existing cardiovascular disease were more likely to be older (mean age 73.7 vs. 67.6 years, *P* < 0.0001), male (55.3% vs. 48.9%, *P* < 0.0001), have a higher burden of comorbid conditions (Charlson comorbidity index score > 1, 85.4% vs. 70.2%, *P* < 0.0001), and advanced stages of lung cancer at diagnosis (stage III and IV, 74.5% vs. 75.6%, *P* < 0.0001). Similarly, they were more likely to have lower neighborhood-level income (< 46 K per year, 36.4% vs. 38.4%, *P* = 0.0237) and education levels (> 80% population attained high-school or greater, 48.9% vs. 51.8%, *P* = 0.0005), and also tended to reside rurally (37.5% vs. 34.6%, *P* < 0.0001). The driving time from place of residence to cancer center (> 2 h, 11.2% vs 11.7%, *P* < 0.0001) was also longer in this subpopulation.

Patients with lung cancer and pre-existing cardiovascular disease were less likely to be treated with surgery (11.2% vs. 18.9%, *P* < 0.0001), chemotherapy (16.4% vs. 32.3%, *P* < 0.0001), or radiotherapy (26.3% vs. 32.1%, *P* < 0.0001). Moreover, such patients were more likely to be treated in community centers rather than academic institutions (51.5% vs. 46.7%, *P* < 0.0001).

### Pre-existing cardiovascular disease and treatment patterns

We used multivariable logistic regression analysis to assess the impact of pre-existing cardiovascular disease on lung cancer treatment, including chemotherapy, radiation therapy, and surgery (Table 3). Adjusting for baseline variables including age, sex, year of diagnosis, Charlson comorbidity index, histological subtype, stage (T, N, and M), laterality, other treatments, management at either academic or community center, zone of residence, driving distance from residence to nearest cancer center, education and income levels, the presence of pre-existing cardiovascular disease was associated with a lower likelihood of chemotherapy (odds ratio, 0.53; 95% confidence interval [CI], 0.48 to 0.58; *P* < 0.0001), radiation therapy (odds ratio, 0.76; 95% CI 0.7 to 0.82, *P* < 0.0001) and surgery (odds ratio, 0.56; 95% CI, 0.44 to 0.70; *P* < 0.0001), respectively.

**Table 3:** Multivariable Logistic regression for factors associated with surgery, chemotherapy, and radiation therapy in patients with lung cancer

Variable	Chemotherapy		Radiation		Surgery	
	Odds Ratio (95% Confidence Limit)	***P*** value	Odds Ratio (95% Confidence Limit)	***P*** value	Odds Ratio (95% Confidence Limit)	***P*** value
**Age group**						
< =60	Reference		Reference		Reference	
61–70	0.68 (0.62 to 0.75)	< 0.0001	0.82 (0.74 to 0.9)	< 0.0001	0.57 (0.44 to 0.75)	< 0.0001
71–80	0.34 (0.30 to 0.37)	< 0.0001	0.61 (0.55 to 0.67)	< 0.0001	0.35 (0.27 to 0.46)	< 0.0001
> 80	0.09 (0.08 to 0.11)	< 0.0001	0.38 (0.34 to 0.43)	< 0.0001	0.12 (0.08 to 0.18)	< 0.0001
**Sex**						
Female	Reference		Reference		Reference	
Male	0.92 (0.85 to 0.99)	0.0225	1.11 (1.04 to 1.18)	0.0029	0.99 (0.82 to 1.21)	0.9448
**Year of diagnosis**	1.01 (1 to 1.02)	0.0337	1.00 (0.99 to 1.01)	0.6327	0.99 (0.96 to 1.02)	0.4833
**CCI score**						
0	Reference		Reference		Reference	
1	0.77 (0.67 to 0.89)	0.0002	1.02 (0.89 to 1.17)	0.7471	0.79 (0.55 to 1.13)	0.1945
> 1	0.68 (0.61 to 0.75)	< 0.0001	1.16 (1.04 to 1.29)	0.0058	0.68 (0.52 to 0.9)	0.0078
**Cardiovascular disease**						
No	Reference		Reference		Reference	
Yes	0.53 (0.48 to 0.58)	< 0.0001	0.76 (0.70 to 0.82)	< 0.0001	0.56 (0.44 to 0.7)	< 0.0001
**Histology**						
Adenocarcinoma	Reference		Reference		Reference	
SCC	0.61 (0.55 to 0.68)	< 0.0001	0.77 (0.71 to 0.85)	< 0.0001	0.12 (0.08 to 0.16)	< 0.0001
SCLC	6.64 (5.93 to 7.45)	< 0.0001	2.17 (1.96 to 2.39)	< 0.0001	0.47 (0.37 to 0.61)	< 0.0001
Carcinoid/LCNE	1.05 (0.84 to 1.31)	0.6779	0.82 (0.62 to 1.08)	0.1480	3.46 (2.16 to 5.54)	< 0.0001
Large cell ca	0.79 (0.58 to 1.08)	0.1382	1.98 (1.45 to 2.71)	< 0.0001	9.65 (4.74 to 19.6)	< 0.0001
Others	6.64 (5.93 to 7.45)	< 0.0001	0.71 (0.63 to 0.79)	< 0.0001	0.48 (0.32 to 0.73)	0.0005
**T stage**						
T0	Reference		Reference			
T1	0.63 (0.37 to 1.09)	0.1007	1.39 (0.78 to 2.48)	0.2709	Reference	
T2	1.02 (0.59 to 1.75)	0.9435	1.74 (0.98 to 3.09)	0.0588	0.78 (0.61 to 1.00)	0.0517
T3	1.28 (0.73 to 2.24)	0.3910	3.52 (1.94 to 6.39)	< 0.0001	0.75 (0.48 to 1.17)	0.2040
T4	1.00 (0.58 to 1.70)	0.9861	1.65 (0.93 to 2.93)	0.0862	0.20 (0.15 to 0.27)	< 0.0001
Unknown	0.74 (0.42 to 1.29)	0.2909	1.06 (0.58 to 1.92)	0.8544	0.97 (0.46 to 2.03)	0.9319
**N stage**						
N0	Reference		Reference		Reference	
N1	2.72 (2.37 to 3.12)	< 0.0001	1.07 (0.93 to 1.24)	0.34	0.97 (0.72 to 1.30)	0.8284
N2	2.22 (1.99 to 2.48)	< 0.0001	1.66 (1.51 to 1.82)	< 0.0001	0.13 (0.1 to 0.17)	< 0.0001
N3	2.36 (2.08 to 2.67)	< 0.0001	1.73 (1.55 to 1.92)	< 0.0001	0.02 (0.01 to 0.03)	< 0.0001
Unknown	1.05 (0.84 to 1.32)	0.6623	0.68 (0.56 to 0.82)	< 0.0001	0.28 (0.15 to 0.51)	< 0.0001
**M stage**						
M0	Reference		Reference		Reference	
M1	0.65 (0.6 to 0.71)	< 0.0001	0.35 (0.32 to 0.37)	< 0.0001	0.03 (0.02 to 0.04)	< 0.0001
Unknown	0.29 (0.18 to 0.45)	< 0.0001	0.28 (0.20 to 0.39)	< 0.0001	1.34 (0.61 to 2.92)	0.4665
**Laterality**						
Bilateral	Reference		Reference		Reference	
Left	0.62 (0.43 to 0.88)	0.0071	3.46 (2.13 to 5.63)	< 0.0001	5.62 (0.69 to 45.6)	0.1063
Right	0.56 (0.39 to 0.79)	0.0012	3.59 (2.21 to 5.83)	< 0.0001	5.63 (0.69 to 45.6)	0.1058
Unknown	0.47 (0.30 to 0.74)	0.0012	1.82 (1.03 to 3.22)	0.0397		
**Chemotherapy**						
0			Reference		Reference	
1			1.21 (1.11 to 1.31)	< 0.0001	1.85 (1.45 to 2.35)	< 0.0001
**Surgery type**						
Segmental	Reference		Reference			
Non-surgery	1.32 (1.13 to 1.55)	0.0006	36.5 (29.3 to 45.48)	< 0.0001		
Pneumonectomy	1.80 (1.34 to 2.41)	< 0.0001	0.43 (0.20 to 0.95)	0.0363		
**Radiation**						
0	Reference				Reference	
1	1.18 (1.08 to 1.28)	0.0001			0.05 (0.04 to 0.07)	< 0.0001
**Immunotherapy**						
0	Reference		Reference		Reference	
1	4.06 (1.53 to 10.8)	0.0050	0.79 (0.29 to 2.1)	0.6303	0.02 (0.00 to 0.31)	0.0045
**Institution type**						
Academic	Reference		Reference		Reference	
Community	0.81 (0.74 to 0.89)	< 0.0001	0.90 (0.83 to 0.98)	0.0097	0.00 (0.00 to 0.01)	< 0.0001
**Driving time (hours)**						
1	Reference		Reference		Reference	
2	0.88 (0.77 to 1.00)	0.0563	0.97 (0.86 to 1.1)	0.6531	1.93 (1.17 to 3.19)	0.0097
> 2	0.92 (0.75 to 1.12)	0.4131	0.90 (0.74 to 1.08)	0.2578	1.05 (0.53 to 2.08)	0.8908
**Health Zone**						
Calgary	Reference		Reference		Reference	
Central	1.36 (1.19 to 1.56)	< 0.0001	0.75 (0.66 to 0.85)	< 0.0001	5.21 (3.26 to 8.34)	< 0.0001
Edmonton	0.97 (0.89 to 1.06)	0.5263	0.99 (0.91 to 1.08)	0.8189	1.90 (1.52 to 2.36)	< 0.0001
North	1.17 (0.95 to 1.44)	0.1448	0.94 (0.77 to 1.15)	0.5443	6.86 (3.40 to 13.85)	< 0.0001
South	1.29 (1.10 to 1.52)	0.0019	0.92 (0.79 to 1.06)	0.2432	2.66 (1.38 to 5.16)	0.0036
**Education level**^a^						
< = 80%	Reference		Reference		Reference	
> 80%	1.06 (0.98 to 1.15)	0.1557	1.00 (0.92 to 1.08)	0.9480	0.94 (0.75 to 1.18)	0.6046
**Income level**						
< = 46 k	Reference		Reference		Reference	
> 46 k	1.21 (1.12 to 1.32)	< 0.0001	1.03 (0.95 to 1.11)	0.4565	1.35 (1.09 to 1.67)	0.0069

*CCI* Charlson’s comorbidity index, *SCC* Squamous cell cancer, *SCLC* Small cell lung cancer, *LCNE* Large Cell Neuroendocrine tumor. ^a^80% of residents have high school and above level of education in the neighborhood

In histology-stratified logistic regression analysis, presence of cardiovascular disease was associated with a lower likelihood of chemotherapy in adenocarcinoma (*P* < 0.0001), squamous cell carcinoma (*P* < 0.0001), small cell lung cancer (*P* < 0.0001), large cell carcinoma (*P* = 0.0157) and other histologies (*P* < 0.0001). However, no association was observed for carcinoid and large cell neuroendocrine tumor (*P* = 0.1275). Similarly, a lower likelihood of radiotherapy was observed in patients with cardiovascular disease and adenocarcinoma (*P* < 0.0001), squamous cell carcinoma (*P* = 0.0009) and other histologies (*P* < 0.0001) but not for small cell lung cancer (*P* = 0.2127), carcinoids (*P* = 4417) and large cell carcinoma (*P* = 0.4259). Lastly, a lower likelihood for surgery was observed for adenocarcinoma (*P* < 0.0001), squamous cell cancer (P < 0.0001), large cell carcinoma (*P* = 0.0046) and other histologies (*P* = 0.0046), but not for small cell lung cancer (*P* = 0.0703) and carcinoids (*P* = 0.6222) (Supplemental Table 1A-C).

In age-stratified logistic regression analyses, presence of cardiovascular disease was associated with a lower likelihood of chemotherapy in patients aged <=60 years (*P* < 0.0001), 61–70 years (*P* < 0.0001), and 71–80 years (*P* < 0.0001) and but not in > 80 years (*P* = 0.0694). A lower likelihood of radiotherapy and surgery was observed in patients with cardiovascular disease across all age groups (Supplemental Table 2A-C).

### Impact of pre-existing cardiovascular disease on survival outcomes

**Fig. 1:**
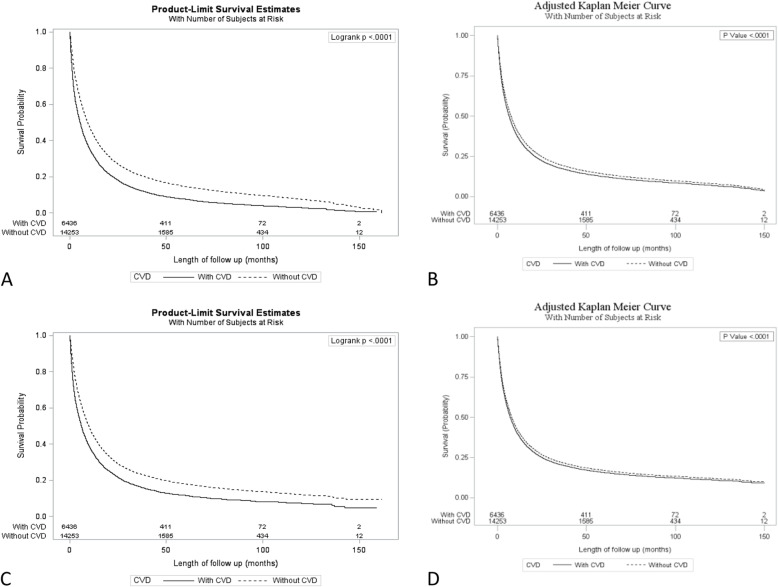
Kaplan-Meier curves showing: **a**, unadjusted overall survival; **b**, adjusted overall survival; **c**, unadjusted cancer specific survival; **d**, adjusted cancer specific survival

The median overall survival (OS) of patients with lung cancer and pre-existing cardiovascular disease was significantly shorter (5.3 [95% CI, 5.1–5.6] months) compared with those without any baseline cardiovascular disease (9.2[95% CI, 8.9–9.6] months, *P* < 0.0001) (Fig. 1). Likewise, cancer specific survival (CSS) was significantly shorter for those with pre-existing cardiovascular disease (6.0 vs 9.8 months, *P* < 0.0001).

A multivariable Cox regression model was constructed to analyze the factors that impacted OS and CSS (Table 4). Adjusting for baseline factors, pre-existing cardiovascular disease was predictive of inferior OS (hazard ratio [HR], 1.1; 95% CI, 1.1 to 1.2; *P* < 0.0001), as well as shorter CSS (HR, 1.1; 95% CI, 1.1 to 1.1; *P* < 0.0001), respectively. Other factors predictive of shorter OS and CSS included older age, male sex, Charlson comorbidity index > 1, histologies other than carcinoid and adenocarcinoma, advanced TNM stage, and > 2 h drive from residence to the nearest cancer center. Conversely, treatment with surgery, chemotherapy, or radiotherapy predicted longer OS and CSS. Notably, treatment setting (academic vs community treatment center), education, and income did not influence survival outcomes. The proportional hazard assumption was verified by plotting the unadjusted OS and CSS curves prior to performing the Cox proportional hazards analysis (Fig. 1a and c). Testing the Schoenfeld residuals for the Cox regression models for OS and CSS showed that the proportional hazards assumption was met.

**Table 4:** Multivariable Cox regression model for factors associated with overall survival and cancer-specific survival in patients with lung cancer

Variable	Overall survival		Cancer specific survival	
	HR (95% Confidence Limit)	***P*** value	HR (95% Confidence Limit)	***P*** value
**Age group**				
< =60	Reference		Reference	
61–70	1.1 (1 to 1.1)	0.0005	1.1 (1 to 1.1)	0.0061
71–80	1.1 (1 to 1.1)	0.0012	1 (1 to 1.1)	0.2039
> 80	1.1 (1.1 to 1.2)	< 0.0001	1.1 (1 to 1.1)	0.0037
**Sex**				
Female	Reference		Reference	
Male	1.2 (1.2 to 1.2)	< 0.0001	1.2 (1.2 to 1.2)	< 0.0001
**Year of diagnosis**	1 (1 to 1)	0.0012	1 (1 to 1)	0.1635
**CCI score**				
0	Reference		Reference	
1	1 (0.9 to 1)	0.2999	1 (0.9 to 1)	0.145
> 1	1.2 (1.1 to 1.2)	< 0.0001	1.2 (1.1 to 1.2)	< 0.0001
**Cardiovascular disease**				
Yes	Reference		Reference	
No	1.1 (1.1 to 1.2)	< 0.0001	1.1 (1.1 to 1.1)	< 0.0001
**Histology**				
Adenocarcinoma	Reference		Reference	
SCC	1.1 (1 to 1.1)	0.0182	1 (1 to 1.1)	0.2506
SCLC	1.6 (1.5 to 1.7)	< 0.0001	1.6 (1.5 to 1.7)	< 0.0001
Carcinoid/LCNE	0.7 (0.7 to 0.8)	< 0.0001	0.8 (0.7 to 0.9)	< 0.0001
Large cell ca	1.3 (1.1 to 1.4)	0.0011	1.3 (1.1 to 1.5)	0.0005
Others	1.2 (1.2 to 1.3)	< 0.0001	1.2 (1.2 to 1.3)	< 0.0001
**T stage**				
T0	Reference		Reference	
T1	1.3 (1 to 1.6)	0.0394	1.1 (0.9 to 1.4)	0.2457
T2	1.8 (1.5 to 2.3)	< 0.0001	1.8 (1.4 to 2.2)	< 0.0001
T3	2.5 (2 to 3.1)	< 0.0001	2.4 (1.9 to 3)	< 0.0001
T4	2.6 (2.1 to 3.2)	< 0.0001	2.5 (2 to 3.1)	< 0.0001
Unknown	1.8 (1.4 to 2.2)	< 0.0001	1.7 (1.4 to 2.2)	< 0.0001
**N stage**				
N0	Reference		Reference	
N1	1.4 (1.3 to 1.5)	< 0.0001	1.5 (1.4 to 1.6)	< 0.0001
N2	1.8 (1.7 to 1.8)	< 0.0001	1.9 (1.8 to 1.9)	< 0.0001
N3	1.8 (1.7 to 1.9)	< 0.0001	1.9 (1.8 to 2)	< 0.0001
Unknown	1.6 (1.5 to 1.7)	< 0.0001	1.7 (1.5 to 1.8)	< 0.0001
**M stage**				
M0	Reference		Reference	
M1	1.9 (1.8 to 1.9)	< 0.0001	2 (1.9 to 2.1)	< 0.0001
Unknown	0.9 (0.8 to 1)	0.2007	0.9 (0.8 to 1.1)	0.2776
**Lateral**				
Bilateral	Reference		Reference	
Left	1.3 (1.1 to 1.5)	0.0001	1.3 (1.1 to 1.5)	0.0014
Right	1.3 (1.2 to 1.5)	< 0.0001	1.3 (1.1 to 1.5)	0.0005
Unknown	2 (1.7 to 2.4)	< 0.0001	1.9 (1.6 to 2.3)	< 0.0001
**Surgery type**				
Pneumonectomy	Reference		Reference	
Lobectomy or segmental	0.7 (0.6 to 0.9)	0.0002	0.6 (0.5 to 0.8)	< 0.0001
Non-surgery	2.8 (2.4 to 3.3)	< 0.0001	2.7 (2.3 to 3.3)	< 0.0001
**Chemotherapy**				
No	Reference		Reference	
Yes	0.4 (0.4 to 0.4)	< 0.0001	0.4 (0.4 to 0.4)	< 0.0001
**Radiation**				
No	Reference		Reference	
Yes	0.6 (0.6 to 0.7)	< 0.0001	0.6 (0.6 to 0.7)	< 0.0001
**Surgery institution type**				
Academic	Reference		Reference	
Community	1 (1 to 1.1)	0.44	1 (1 to 1.1)	0.069
**Driving time (hours)**				
1	Reference		Reference	
2	1 (1 to 1.1)	0.5613	1 (1 to 1.1)	0.4693
3	0.9 (0.8 to 1)	0.0215	0.9 (0.8 to 1)	0.0073
**Zone name**				
Calgary	Reference		Reference	
Central	1.1 (1 to 1.1)	0.004	1.1 (1 to 1.1)	0.03
Edmonton	1.1 (1 to 1.1)	< 0.0001	1.1 (1.1 to 1.1)	< 0.0001
North	1.2 (1.1 to 1.3)	< 0.0001	1.2 (1.1 to 1.3)	< 0.0001
South	1.2 (1.1 to 1.3)	< 0.0001	1.2 (1.1 to 1.3)	< 0.0001
**Education level**^a^				
< = 80%	Reference		Reference	
> 80%	1 (1 to 1)	0.3769	1 (1 to 1)	0.5999
**Income level**				
< = 46 k	Reference		Reference	
> 46 k	1 (1 to 1)	0.9992	1 (1 to 1)	0.8051

*CCI* Charlson’s comorbidity index, *SCC* Squamous cell cancer, *SCLC* Small cell lung cancer, *LCNE* Large Cell Neuroendocrine tumor. ^a^80% of residents have high school and above level of education in the neighborhood

Therefore, we performed age-stratified Cox regression analyses for age groups: ≤ 60 years, 61–70 years, 71–80 years and > 80 years. Presence of baseline cardiovascular disease predicted worse CSS (≤ 60 years: HR, 1.01; 95% CI, 1.00 to 1.02; *P* = 0.0489; 61–70 years: HR, 1.11; 95% CI, 1.04 to 1.19; *P* = 0.0017; 71–80 years: HR, 1.08; 95% CI, 1.02 to 1.14; *P* = 0.0099; > 80 years: HR, 1.11; 95% CI, 1.03 to 1.20; *P* = 0.0067) as well as OS (< 60 years: HR, 1.14; 95% CI, 1.04 to 1.25; *P* = 0.0059; 61–70 years: HR, 1.16; 95% CI, 1.08 to 1.23; *P* < 0.0001; 71–80 years: HR, 1.14; 95% CI, 1.08 to 1.20; *P* < 0.0001; > 80 years: HR, 1.15; 95% CI, 1.07 to 1.23; *P* < 0.0001) in all age groups.

The presence of pre-existing cardiovascular disease can alter the effect of various treatments on the outcomes of patients with lung cancer. Therefore, we assessed for any effect modification by examining interactions in the statistical models. The effect of baseline cardiovascular disease on OS varied for chemotherapy (*P* = 0.026), surgery (*P* < 0.0001), and radiotherapy (*P* = 0.001). Likewise, for CSS, the interactions were significant for radiotherapy and surgery (*P* = 0.050, and *P* = 0.001, respectively). However, the interaction of chemotherapy on CSS showed only a trend towards significance (*P* = 0.060).

Finally, in the competing risk multistate model adjusted for baseline characteristics, we found that presence of cardiovascular disease was associated with an increased risk of non-cancer related death (HR, 1.48; 95% CI, 1.33–1.64; *P* < 0.0001) but not cancer related death (HR, 0.98; 95% CI, 0.94–1.03; *P* = 0.460) (Table 5).

**Table 5:** Competing risk multistate model

Variable	Cancer related death		Non-cancer related death	
	HR (95% Confidence Limit)	***P*** value	HR (95% Confidence Limit)	***P*** value
**Age group**				
< =60	Reference			
61–70	1.05 (1.00–1.10)	0.074	1.24 (1.05–1.46)	0.010
71–80	1.01 (0.96–1.06)	0.760	1.60 (1.36–1.88)	< 0.0001
> 80	1.07 (1.00–1.14)	0.037	1.34 (1.10–1.63)	0.004
**Sex**				
Male	Reference			
Female	0.89 (0.85–0.92)	< 0.0001	0.95 (0.87–1.05)	0.340
**Year of diagnosis**	0.98 (0.98–0.99)	< 0.0001	1.06 (1.05–1.08)	< 0.0001
**CCI score**				
0	Reference			
1	0.95 (0.89–1.02)	0.140	1.07 (0.88–1.31)	0.480
2	1.11 (1.05–1.17)	0.001	1.10 (0.94–1.30)	0.250
**CVD**				
Without CVD	Reference			
With CVD	0.98 (0.94–1.03)	0.460	1.48 (1.33–1.64)	< 0.0001
**Grade**				
1	Reference			
2	1.43 (1.25–1.63)	< 0.0001	1.18 (0.91–1.53)	0.220
3	1.71 (1.51–1.94)	< 0.0001	1.02 (0.78–1.34)	0.870
Unknown	1.53 (1.35–1.73)	< 0.0001	1.10 (0.84–1.44)	0.480
**Histology**				
Adenocarcinoma	Reference			
Carcinoid/LCNE	0.83 (0.72–0.96)	0.011	0.83 (0.59–1.167)	0.280
Large cell ca	1.19 (1.01–1.41)	0.036	0.80 (0.49–1.321)	0.390
Others	1.18 (1.12–1.24)	< 0.0001	0.83 (0.72–0.949)	0.007
SCC	1.00 (0.96–1.06)	0.780	1.16 (1.02–1.323)	0.030
SCLC	1.40 (1.31–1.49)	< 0.0001	0.96 (0.79–1.17)	0.700
**T stage**				
T0	Reference			
T1	1.06 (0.79–1.42)	0.680	1.32 (0.50–3.47)	0.570
T2	1.54 (1.16–2.06)	0.003	1.06 (0.40–2.77)	0.910
T3	1.94 (1.44–2.62)	< 0.0001	0.86 (0.32–2.32)	0.760
T4	2.04 (1.53–2.72)	< 0.0001	0.78 (0.30–2.05)	0.620
Unknown	1.48 (1.10–1.99)	0.010	1.14 (0.43–2.99)	0.800
**N stage**				
N0	Reference			
N1	1.38 (1.29–1.48)	< 0.0001	0.80 (0.67–0.95)	0.011
N2	1.69 (1.61–1.78)	< 0.0001	0.53 (0.46–0.61)	< 0.0001
N3	1.73 (1.63–1.84)	< 0.0001	0.56 (0.46–0.67)	< 0.0001
Unknown	1.45 (1.31–1.60)	< 0.0001	0.87 (0.68–1.11)	0.250
**M stage**				
M0	Reference			
M1	1.88 (1.80–1.96)	< 0.0001	0.46 (0.40–0.52)	< 0.0001
Unknown	0.89 (0.76–1.04)	0.160	1.13 (0.79–1.62)	0.510
**Lateral**				
Bilateral	Reference			
Left	1.05 (0.88–1.26)	0.570	1.06 (0.56–1.99)	0.870
Right	1.06 (0.89–1.28)	0.490	0.99 (0.53–1.88)	0.990
Unknown	1.52 (1.21–1.90)	< 0.0001	0.97 (0.45–2.10)	0.940
**Surgery type**				
No surgery	Reference			
Lobectomy or segmental	0.32 (0.30–0.35)	< 0.0001	0.72 (0.59–0.88)	0.002
Pneumonectomy	0.48 (0.40–0.57)	< 0.0001	0.73 (0.48–1.13)	0.160
**Chemotherapy**				
0	Reference			
1	0.47 (0.45–0.49)	< 0.0001	1.14 (1.00–1.30)	0.047
**Radiation**				
0	Reference			
1	0.74 (0.71–0.77)	< 0.0001	0.90 (0.80–1.02)	0.110
**Immunotherapy**				
0	Reference			
1	0.52 (0.32–0.85)	0.010	1.44 (0.33–6.31)	0.620
**Surgery institution type**				
Academic	Reference			
Community	1.07 (1.03–1.12)	0.002	0.72 (0.63–0.83)	< 0.0001
**Driving time to the nearest cancer center (hours)**				
1	Reference			
2	1.06 (0.99–1.13)	0.074	0.94 (0.79–1.11)	0.440
> 2	0.88 (0.79–0.97)	0.013	1.15 (0.89–1.49)	0.290
**Zone name**				
Calgary	Reference			
Central	1.02 (0.95–1.09)	0.640	1.22 (1.02–1.46)	0.030
Edmonton	1.09 (1.04–1.14)	0.001	0.84 (0.74–0.96)	0.009
North	1.19 (1.07–1.32)	0.001	1.02 (0.77–1.34)	0.910
South	1.10 (1.02–1.20)	0.019	1.15 (0.93–1.43)	0.190
**Educational level**^a^				
< = 80%	Reference			
> 80%	1.00 (0.96–1.04)	0.950	0.96 (0.86–1.07)	0.480
**Income level**				
< = 46 k	Reference			
> 46 k	1.02 (0.98–1.06)	0.430	0.92 (0.82–1.03)	0.160

*CCI* Charlson’s comorbidity index, *SCC* Squamous cell cancer, *SCLC* Small cell lung cancer, *LCNE* Large Cell Neuroendocrine tumor. ^a^80% of residents have high school and above level of education in the neighborhood

## Discussion

In this large study of over 20,000 patients that included patients across all stages of lung cancer, approximately one-third of patients had pre-existing cardiovascular disease, most commonly congestive heart failure. The presence of baseline heart disease was associated with a lower likelihood of receiving any form of cancer treatment including chemotherapy, radiotherapy or surgery. Further, pre-existing cardiovascular disease in patients with lung cancer predicted for inferior survival OS and CSS. Presence of cardiovascular disease was associated with increased non-cancer related deaths but not cancer related deaths in the multistate model. This suggests that the excess mortality in patients with cardiovascular disease and lung cancer was related to the increased risk of non-cancer related deaths.

A previous study from the United States linking the Surveillance, Epidemiology, and End Results data and Medicare (SEER) data reported on the effect of cardiovascular disease on survival outcomes in patients with non-small cell lung cancer. Ischemic heart disease (33.7%), cardiac arrythmias (28.6%), and congestive heart failure (17.5%) were the common cardiovascular diseases reported in that study [23]. The corresponding prevalence of these conditions in our study were lower at 10.3, 9.4, and 14.7%, respectively. One potential reason for this difference is that the age group of patients across studies were remarkably different. While older age was not an exclusion criterion and 21.5% of patients were less than 60 years old in the current study, those less than 65 years were excluded from the study in the United States. Moreover, a lower frequency of obesity and cigarette smoking in Canadians as compared with Americans may be another factor contributing to the observed differences in cardiovascular diseases [26, 27].

The prevalence of baseline cardiovascular disease in patients with breast cancer was as low as 8% in a prior Canadian study [21]. However, that study included women exclusively and the median age of patients was 59 years. The higher prevalence of cardiovascular disease in this study may reflect the larger proportion of older males in the current study cohort [28]. Notably, the pattern of cardiovascular diseases was also different. For instance, cerebrovascular accidents (2.6%) and cardiac arrythmias (2.0%) were more common than heart failure (1.8%) and myocardial infarction (1.1%) in women with breast cancer. Conversely, heart failure (14.7%) and cerebrovascular accidents (12.5%) were more prevalent than myocardial infarction (10.3%) and cardiac arrythmias (9.4%) in patients with lung cancer. The drivers of these differences are likely multifactorial, but one factor could be the role of estrogen in preventing ischemic heart disease in younger women. Another reason could be differences in modifiable risk factors, such as smoking and obesity, between men and women [28, 29].

As expected, patients with baseline cardiovascular disease were less likely to receive any form of treatment for lung cancer. This finding was observed across all age groups, except for chemotherapy in patients older than 80 years. It is likely that a proportion of these patients were considered unfit to receive therapy, in which case their lack of treatment is clinically appropriate. However, we hypothesized that others may have been denied treatment because of perceived concerns that their cardiovascular comorbidity may place them at heightened risk of toxicities or because of the absence of data from clinical trials regarding the safety of chemotherapy administration in patients with heart disease [30]. It has been reported that heart disease represents the second most common cause of ineligibility for clinical trials [31, 32]. This results in limited data to guide oncologists when they treat comorbid patients in the real-world and contributes to variations in management whereby physicians base their decisions on anecdotal experience. It is likely in these scenarios that physicians resort to the use of patient-related factors such as age and performance status, as well as the expertise of cardio-oncologists in formulating their treatment plans [33, 34]. Broadening of eligibility criteria in clinical trials to represent patients with a history of cardiovascular disease will increase the generalizability of results to real-world patients and optimize evidence-based treatment in this subpopulation.

Our study found that the OS of patients with lung cancer who had baseline cardiovascular disease were worse compared to those without pre-existing cardiac comorbid conditions. Similar findings were reported in the SEER and Medicare linked study from the United States of older beneficiaries [23]. Another small study of 247 patients with limited stage lung cancer also showed increased postoperative morbidity and mortality among patients with lung cancer and pre-existing cardiovascular disease [22]. Importantly, inferior OS were also reported in a large Canadian study of patients with breast cancer and baseline cardiovascular disease, suggesting that this observation may persist across different tumor types [21].

Interestingly, we found that prior cardiovascular disease was associated with an increased risk of non-cancer related death but not cancer related death in the competing risk multistate model. However, patients with cardiovascular disease had worse CSS when competing risks were not used in the Cox model. This finding highlights the previously reported overestimation of cancer-specific deaths in time-to-event analysis where non-cancer specific deaths are censored, especially among patients with comorbidities. In such models, patients who die of unrelated causes are censored at death and wrongly assumed to have had the same probability of cancer related death compared to those who continue with follow-up [35]. Therefore, a competing risk model is generally considered more appropriate when patients have other competing risks of death. This is particularly true for patients with lung cancer who are likely to be older and may be afflicted with other pulmonary or cardiac conditions that can represent a serious threat to their overall health [19, 20].

The management of lung cancer is experiencing a significant paradigm shift with the recent approvals of various targeted therapies and immune checkpoint inhibitors, all of which have demonstrated improved survival across different subsets of patients with lung cancer [36–40]. In this study, patients with pre-existing cardiovascular disease were less likely to receive immunotherapy, although only 20 patients received checkpoint inhibitors in our cohort. The use of novel immunotherapeutic agents in patients with lung cancer is increasing after they were approved in Canada in 2016 [41, 42]. Since one-third of patients with lung cancer were found to have associated cardiovascular disease, it is imperative that early cardio-oncology consultations be sought in such patients to optimize the concurrent management of comorbid conditions so that the benefit of novel therapies can be extended to these patients in the real-world.

The study was limited by its retrospective design and use of administrative sources for data. Therefore, the clinical severity of cardiovascular disease and performance status could not be ascertained reliably. Further, data on other associated characteristics, such as smoking and body mass index, were not available. These factors could potentially act as confounders and it may be possible that the treatment patterns and survival outcomes in patients with lung cancer could vary based on such factors. Lastly, the presence of cardiovascular disease was assumed as the reason for not receiving treatment, even though additional factors such as patient preference and physician discretion are major contributors to treatment decisions. However, the large sample size of our study and the population-based nature of the data are major strengths.

## Conclusions

In summary, a significant proportion of patients with lung cancer have baseline cardiovascular disease, which is associated with a lower likelihood of receiving any cancer treatment and also a higher risk of cancer-related death. With a plethora of novel therapies being developed, early engagement and co-management with experts in cardio-oncology can help to optimize the number of lung cancer patients who can derive benefit from emerging therapies.

## Supplementary information

**Additional file 1: Supplemental Table 1.** Histology stratified logistic regression to predict the likelihood of treatment (A: Chemotherapy, B: Radiotherapy, C: Surgery). **Supplemental Table 2.** Age stratified logistic regression analysis to predict the likelihood of treatment (A: Chemotherapy, B: Radiotherapy, C: Surgery).

## Data Availability

The datasets used and/or analysed during the current study are available from the corresponding author on reasonable request.

## Abbreviations

ACR: Alberta Cancer Registry
ICD-10: International Classification of Diseases tenth version
OS: Overall Survival
CSS: Cancer Specific Survival
CI: Confidence Interval
OR: Odds Ratio
HR: HR
