# Supplementary material for: Impact of pre-existing cardiovascular disease on treatment patterns and survival outcomes in patients with lung cancer
Source: BMC Cancer. 2020 Oct 15;20:1004. doi: 10.1186/s12885-020-07487-9 (PMC7559447; doi:10.1186/s12885-020-07487-9)
Supplement: Supplementary file 1 — Additional file 1: Supplemental Table 1. Histology stratified logistic regression to predict the likelihood of treatment (A: Chemotherapy, B: Radiotherapy, C: Surgery). Supplemental Table 2. Age stratified logistic regression analysis to predict the likelihood of treatment (A: Chemotherapy, B: Radiotherapy, C: Surgery). [file 12885_2020_7487_MOESM1_ESM.docx]

**Supplemental Table 1:**

**Histology stratified logistic regression to predict the likelihood of treatment (A: Chemotherapy, B: Radiotherapy, C: Surgery)**

A

|  |  | **Adenocarcinoma** | | **Squamous cell cancer** | | **Small cell lung cancer** | | **Carcinoid/Large cell neuroendocrine cancer** | | **Large cell carcinoma** | | **Others** | |
| --- | --- | --- | --- | --- | --- | --- | --- | --- | --- | --- | --- | --- | --- |
| **Variable** | **Category** | **Odds Ratio (95% Confidence Limit)** | **P value** | **Odds Ratio (95% Confidence Limit)** | **P value** | **Odds Ratio (95% Confidence Limit)** | **P value** | **Odds Ratio (95% Confidence Limit)** | **P value** | **Odds Ratio (95% Confidence Limit)** | **P value** | **Odds Ratio (95% Confidence Limit)** | **P value** |
| Age group | <=60 | Reference |  | Reference |  | Reference |  | Reference |  | Reference |  | Reference |  |
|  | 61-70 | 0.66 (0.58 to 0.76) | <.0001 | 0.7 (0.56 to 0.88) | 0.0021 | 0.79 (0.6 to 1.03) | 0.0786 | 0.84 (0.48 to 1.47) | 0.5353 | 0.87 (0.4 to 1.92) | 0.7324 | 0.61 (0.49 to 0.74) | <.0001 |
|  | >70 | 0.27 (0.23 to 0.31) | <.0001 | 0.24 (0.19 to 0.31) | <.0001 | 0.38 (0.3 to 0.49) | <.0001 | 0.4 (0.22 to 0.73) | 0.0027 | 0.21 (0.08 to 0.54) | 0.0011 | 0.15 (0.12 to 0.19) | <.0001 |
| Sex | Female | Reference |  | Reference |  | Reference |  | Reference |  | Reference |  | Reference |  |
|  | Male | 0.92 (0.83 to 1.03) | 0.1601 | 0.98 (0.81 to 1.17) | 0.7973 | 0.92 (0.77 to 1.11) | 0.3823 | 1.06 (0.67 to 1.68) | 0.798 | 0.64 (0.32 to 1.28) | 0.2095 | 0.89 (0.75 to 1.05) | 0.1685 |
| Year of diagnosis | Continuous | 1.02 (1 to 1.04) | 0.0165 | 1 (0.97 to 1.03) | 0.9859 | 1 (0.97 to 1.02) | 0.8125 | 0.94 (0.88 to 1.01) | 0.1036 | 0.92 (0.83 to 1.03) | 0.1667 | 1.01 (0.99 to 1.04) | 0.3872 |
| CCI score | 0 | Reference |  | Reference |  | Reference |  | Reference |  | Reference |  | Reference |  |
|  | 1 | 0.73 (0.59 to 0.89) | 0.0024 | 0.91 (0.64 to 1.29) | 0.5949 | 0.99 (0.63 to 1.56) | 0.9748 | 1.07 (0.43 to 2.64) | 0.891 | 1.61 (0.39 to 6.66) | 0.5133 | 0.77 (0.55 to 1.06) | 0.1115 |
|  | 2 | 0.57 (0.49 to 0.66) | <.0001 | 0.68 (0.52 to 0.89) | 0.0049 | 0.76 (0.54 to 1.06) | 0.1034 | 1.2 (0.59 to 2.42) | 0.6138 | 1.33 (0.4 to 4.45) | 0.6429 | 0.98 (0.77 to 1.26) | 0.8868 |
| CVD | Without CVD | Reference |  | Reference |  | Reference |  | Reference |  | Reference |  | Reference |  |
|  | With CVD | 0.52 (0.46 to 0.6) | <.0001 | 0.43 (0.34 to 0.54) | <.0001 | 0.6 (0.49 to 0.73) | <.0001 | 0.64 (0.36 to 1.13) | 0.1275 | 0.28 (0.1 to 0.78) | 0.0157 | 0.42 (0.34 to 0.53) | <.0001 |
| Stage | I | Reference |  | Reference |  | Reference |  | Reference |  | Reference |  | Reference |  |
|  | II | 14.79 (10.94 to 19.99) | <.0001 | 8.56 (5.7 to 12.86) | <.0001 | 1.51 (0.57 to 4.01) | 0.4048 | 2.04 (0.75 to 5.56) | 0.1622 | 7.41 (1.88 to 29.16) | 0.0042 | 6.06 (3.03 to 12.13) | <.0001 |
|  | III | 14.26 (11.08 to 18.37) | <.0001 | 11.75 (7.85 to 17.58) | <.0001 | 1.21 (0.68 to 2.16) | 0.5155 | 4.57 (1.81 to 11.55) | 0.0013 | 8.51 (2.28 to 31.79) | 0.0015 | 7.71 (4.58 to 12.97) | <.0001 |
|  | IV | 7.66 (5.89 to 9.96) | <.0001 | 6.92 (4.54 to 10.55) | <.0001 | 0.74 (0.42 to 1.31) | 0.3062 | 4 (1.52 to 10.5) | 0.0049 | 2.1 (0.48 to 9.14) | 0.3226 | 5.06 (3.04 to 8.44) | <.0001 |
|  | UNK | 2.57 (1.21 to 5.45) | 0.0141 | 1.89 (0.62 to 5.76) | 0.2651 | 0.24 (0.09 to 0.63) | 0.0038 | 0.06 (0.01 to 0.48) | 0.0079 | Reference |  | 2.97 (1.49 to 5.95) | 0.0021 |
| Surgery type | lobectomy or segmental | Reference |  | Reference |  | Reference |  | Reference |  | 0.52 (0.12 to 2.32) | 0.3932 | Reference |  |
|  | no surgery | 0.82 (0.66 to 1.02) | 0.0741 | 0.41 (0.28 to 0.59) | <.0001 | 0.72 (0.35 to 1.49) | 0.3775 | 2.28 (0.87 to 5.95) | 0.0926 | 1.03 (0.24 to 4.45) | 0.9698 | 0.22 (0.12 to 0.4) | <.0001 |
|  | pneumonectomy | 0.79 (0.49 to 1.28) | 0.3418 | 1.7 (1.03 to 2.8) | 0.038 | 0.13 (0.01 to 1.66) | 0.1154 | 1.11 (0.22 to 5.63) | 0.901 | Reference |  | 0.76 (0.24 to 2.46) | 0.653 |
| Radiation | 0 | Reference |  | Reference |  | Reference |  | Reference |  | 1.01 (0.44 to 2.34) | 0.98 | Reference |  |
|  | 1 | 0.82 (0.72 to 0.93) | 0.0026 | 1.24 (1 to 1.53) | 0.0485 | 3.38 (2.64 to 4.32) | <.0001 | 1.08 (0.6 to 1.96) | 0.7982 | Reference |  | 1.55 (1.29 to 1.86) | <.0001 |
| Surgery institution type | academic | Reference |  | Reference |  | Reference |  | Reference |  | 0.81 (0.3 to 2.15) | 0.6687 | Reference |  |
|  | community | 0.84 (0.73 to 0.96) | 0.0127 | 0.87 (0.69 to 1.09) | 0.2181 | 0.75 (0.6 to 0.93) | 0.0077 | 0.55 (0.3 to 1.01) | 0.0525 | Reference |  | 0.69 (0.56 to 0.84) | 0.0003 |
| Driving time to the nearest cancer center (minutes) | 1 | Reference |  | Reference |  | Reference |  | Reference |  | 1.56 (0.41 to 5.93) | 0.5124 | Reference |  |
|  | 2 | 0.87 (0.71 to 1.07) | 0.2005 | 1.07 (0.78 to 1.47) | 0.6583 | 0.99 (0.7 to 1.4) | 0.9569 | 1.3 (0.53 to 3.15) | 0.5646 | 2.62 (0.27 to 25.61) | 0.4082 | 0.73 (0.53 to 1) | 0.0511 |
|  | 3 | 0.91 (0.66 to 1.25) | 0.5471 | 1.18 (0.73 to 1.93) | 0.5011 | 0.8 (0.48 to 1.34) | 0.3978 | 0.94 (0.28 to 3.2) | 0.9261 | Reference |  | 0.78 (0.51 to 1.2) | 0.2542 |
| Zone name | Calgary | Reference |  | Reference |  | Reference |  | Reference |  | 1.61 (0.34 to 7.61) | 0.5455 | Reference |  |
|  | Central | 1.39 (1.13 to 1.7) | 0.0017 | 1.27 (0.91 to 1.77) | 0.161 | 0.99 (0.7 to 1.42) | 0.9707 | 2.28 (1.01 to 5.14) | 0.0473 | 1.29 (0.54 to 3.09) | 0.5723 | 2.02 (1.44 to 2.83) | <.0001 |
|  | Edmonton | 1.07 (0.94 to 1.23) | 0.3148 | 0.93 (0.74 to 1.17) | 0.5167 | 0.59 (0.47 to 0.75) | <.0001 | 1.25 (0.7 to 2.24) | 0.4477 | 0.83 (0.08 to 8.17) | 0.8715 | 1.26 (1 to 1.59) | 0.0475 |
|  | North | 1.11 (0.79 to 1.55) | 0.5423 | 0.9 (0.53 to 1.51) | 0.6883 | 1.01 (0.58 to 1.74) | 0.9795 | 1.31 (0.36 to 4.77) | 0.678 | 3.83 (0.91 to 16.14) | 0.0675 | 1.87 (1.17 to 3.01) | 0.0095 |
|  | South | 1.3 (1.02 to 1.66) | 0.0366 | 1.01 (0.69 to 1.47) | 0.9611 | 0.98 (0.63 to 1.5) | 0.9114 | 1.07 (0.34 to 3.34) | 0.9103 | Reference |  | 1.7 (1.11 to 2.6) | 0.0143 |
| Educational attainment of neighborhood (% high school or greater) | <= 80% | Reference |  | Reference |  | Reference |  | Reference |  | 1.65 (0.77 to 3.51) | 0.1975 | Reference |  |
|  | > 80% | 1.13 (1 to 1.29) | 0.0539 | 1.05 (0.86 to 1.29) | 0.6144 | 0.92 (0.75 to 1.13) | 0.4535 | 0.85 (0.5 to 1.42) | 0.5309 | Reference |  | 1 (0.83 to 1.21) | 0.9868 |
| Median neighborhood income | <= 46k | Reference |  | Reference |  | Reference |  | Reference |  | 1.57 (0.75 to 3.29) | 0.23 | Reference |  |
|  | > 46k | 1.23 (1.09 to 1.4) | 0.0009 | 1.06 (0.86 to 1.31) | 0.5883 | 1.18 (0.96 to 1.46) | 0.1187 | 1.02 (0.6 to 1.71) | 0.9528 |  |  | 1.2 (0.99 to 1.45) | 0.0646 |

**B**

|  |  | **Adenocarcinoma** | | **Squamous cell cancer** | | **Small Cell Lung Cancer** | | **Carcinoid/Large cell neuroendocrine** | | **Large cell carcinoma** | | **Others** | |
| --- | --- | --- | --- | --- | --- | --- | --- | --- | --- | --- | --- | --- | --- |
| **Variable** | **Category** | **Odds Ratio (95% Confidence Limit)** | **P value** | **Odds Ratio (95% Confidence Limit)** | **P value** | **Odds Ratio (95% Confidence Limit)** | **P value** | **Odds Ratio (95% Confidence Limit)** | **P value** | **Odds Ratio (95% Confidence Limit)** | **P value** | **Odds Ratio (95% Confidence Limit)** | **P value** |
| Age group | <=60 | Reference |  | Reference |  | Reference |  | Reference |  | Reference |  | Reference |  |
|  | 61-70 | 0.92 (0.79 to 1.06) | 0.2331 | 0.66 (0.52 to 0.85) | 0.0013 | 0.91 (0.72 to 1.15) | 0.4243 | 0.61 (0.29 to 1.29) | 0.1957 | 1.3 (0.52 to 3.24) | 0.5733 | 0.65 (0.54 to 0.79) | <.0001 |
|  | 71-80 | 0.67 (0.57 to 0.78) | <.0001 | 0.63 (0.49 to 0.81) | 0.0003 | 0.56 (0.43 to 0.72) | <.0001 | 0.66 (0.31 to 1.42) | 0.2884 | 1 (0.4 to 2.5) | 0.9992 | 0.43 (0.35 to 0.51) | <.0001 |
|  | >80 | 0.47 (0.38 to 0.57) | <.0001 | 0.54 (0.4 to 0.73) | <.0001 | 0.34 (0.22 to 0.53) | <.0001 | 0.58 (0.21 to 1.61) | 0.2952 | 0.29 (0.09 to 0.97) | 0.0447 | 0.2 (0.16 to 0.25) | <.0001 |
| Sex | Female | Reference |  | Reference |  | Reference |  | Reference |  | Reference |  | Reference |  |
|  | Male | 1.01 (0.9 to 1.12) | 0.9058 | 1.29 (1.1 to 1.52) | 0.0015 | 1.17 (0.97 to 1.42) | 0.0984 | 0.97 (0.55 to 1.71) | 0.9148 | 0.99 (0.49 to 1.99) | 0.981 | 1.19 (1.05 to 1.34) | 0.007 |
| Year of diagnosis | Continuous | 0.99 (0.97 to 1) | 0.0783 | 1 (0.98 to 1.03) | 0.7279 | 1.04 (1.01 to 1.07) | 0.005 | 0.88 (0.81 to 0.96) | 0.0058 | 1.05 (0.93 to 1.17) | 0.4503 | 0.99 (0.97 to 1.01) | 0.1853 |
| CCI score | 0 | Reference |  | Reference |  | Reference |  | Reference |  | Reference |  | Reference |  |
|  | 1 | 1.02 (0.81 to 1.28) | 0.8493 | 1.13 (0.8 to 1.61) | 0.4839 | 0.8 (0.53 to 1.22) | 0.3082 | 1.43 (0.46 to 4.46) | 0.5333 | 0.64 (0.15 to 2.78) | 0.5509 | 1.08 (0.84 to 1.38) | 0.5424 |
|  | 2 | 1.15 (0.98 to 1.36) | 0.0893 | 0.91 (0.7 to 1.2) | 0.5163 | 0.96 (0.71 to 1.31) | 0.8164 | 1.21 (0.51 to 2.89) | 0.6631 | 0.5 (0.16 to 1.54) | 0.2237 | 1.57 (1.28 to 1.92) | <.0001 |
| CVD | Without CVD | Reference |  | Reference |  | Reference |  | Reference |  | Reference |  | Reference |  |
|  | With CVD | 0.71 (0.62 to 0.81) | <.0001 | 0.75 (0.63 to 0.89) | 0.0009 | 0.87 (0.7 to 1.08) | 0.2127 | 0.77 (0.39 to 1.51) | 0.4417 | 0.72 (0.32 to 1.62) | 0.4259 | 0.7 (0.61 to 0.81) | <.0001 |
| Stage | I-II | Reference |  | Reference |  | Reference |  | Reference |  | Reference |  | Reference |  |
|  | III | 1 (0.81 to 1.24) | 0.9919 | 1.3 (1.03 to 1.66) | 0.0286 | 1.04 (0.67 to 1.6) | 0.8637 | 6.09 (1.8 to 20.58) | 0.0036 | 1.08 (0.25 to 4.7) | 0.9135 | 1.06 (0.87 to 1.29) | 0.5524 |
|  | IV | 0.37 (0.3 to 0.45) | <.0001 | 0.44 (0.35 to 0.56) | <.0001 | 0.19 (0.13 to 0.3) | <.0001 | 2.14 (0.65 to 7.04) | 0.2106 | 0.2 (0.05 to 0.83) | 0.0269 | 0.47 (0.39 to 0.57) | <.0001 |
|  | UNK | 0.2 (0.11 to 0.36) | <.0001 | 0.17 (0.09 to 0.3) | <.0001 | 0.59 (0.21 to 1.61) | 0.3016 | 0.59 (0.12 to 2.91) | 0.5184 | Reference |  | 0.2 (0.13 to 0.32) | <.0001 |
| Surgery type | no surgery | Reference |  | Reference |  | Reference |  | Reference |  | 0.01 (0 to 0.05) | <.0001 | Reference |  |
|  | had surgery | 0.03 (0.02 to 0.03) | <.0001 | 0.01 (0.01 to 0.02) | <.0001 | 0.1 (0.05 to 0.23) | <.0001 | 0.06 (0.02 to 0.21) | <.0001 | Reference |  | 0.14 (0.07 to 0.27) | <.0001 |
| Chemo | 0 | Reference |  | Reference |  | Reference |  | Reference |  | 1.05 (0.43 to 2.55) | 0.9172 | Reference |  |
|  | 1 | 0.85 (0.75 to 0.97) | 0.0139 | 1.33 (1.08 to 1.65) | 0.0078 | 3.19 (2.49 to 4.09) | <.0001 | 1.05 (0.57 to 1.91) | 0.883 | Reference |  | 1.55 (1.29 to 1.85) | <.0001 |
| Surgery institution type | academic | Reference |  | Reference |  | Reference |  | Reference |  | 0.9 (0.38 to 2.13) | 0.8109 | Reference |  |
|  | community | 0.9 (0.79 to 1.03) | 0.1176 | 0.89 (0.74 to 1.08) | 0.2394 | 1.08 (0.87 to 1.36) | 0.4749 | 0.62 (0.32 to 1.2) | 0.1553 | Reference |  | 0.89 (0.77 to 1.03) | 0.1045 |
| Driving time to the nearest cancer center (minutes) | 1 | Reference |  | Reference |  | Reference |  | Reference |  | 0.64 (0.19 to 2.13) | 0.464 | Reference |  |
|  | 2 | 1.02 (0.83 to 1.25) | 0.8441 | 1.24 (0.95 to 1.62) | 0.1103 | 0.99 (0.68 to 1.43) | 0.944 | 2.04 (0.68 to 6.12) | 0.2049 | 0.17 (0.02 to 1.38) | 0.0971 | 0.8 (0.63 to 1.01) | 0.0588 |
|  | 3 | 0.95 (0.68 to 1.31) | 0.7419 | 1.11 (0.71 to 1.73) | 0.6416 | 1 (0.58 to 1.73) | 0.9999 | 1.35 (0.28 to 6.45) | 0.7047 | Reference |  | 0.76 (0.54 to 1.06) | 0.1052 |
| Zone name | Calgary | Reference |  | Reference |  | Reference |  | Reference |  | 0.97 (0.24 to 3.99) | 0.9655 | Reference |  |
|  | Central | 0.84 (0.68 to 1.04) | 0.1063 | 0.64 (0.48 to 0.86) | 0.0026 | 0.85 (0.58 to 1.24) | 0.4074 | 0.71 (0.24 to 2.12) | 0.5413 | 0.87 (0.36 to 2.11) | 0.765 | 0.73 (0.57 to 0.93) | 0.0116 |
|  | Edmonton | 1.02 (0.89 to 1.17) | 0.7648 | 0.83 (0.68 to 1.01) | 0.0625 | 1.6 (1.25 to 2.05) | 0.0002 | 1.36 (0.67 to 2.74) | 0.391 | 1.22 (0.17 to 8.87) | 0.8434 | 1.09 (0.93 to 1.27) | 0.2904 |
|  | North | 0.97 (0.69 to 1.36) | 0.8613 | 0.63 (0.4 to 1.01) | 0.0534 | 1.41 (0.79 to 2.53) | 0.2408 | 0.92 (0.19 to 4.55) | 0.9232 | 0.88 (0.25 to 3.1) | 0.8375 | 1.06 (0.74 to 1.52) | 0.7374 |
|  | South | 1.09 (0.86 to 1.38) | 0.4963 | 0.71 (0.52 to 0.97) | 0.0298 | 1.1 (0.71 to 1.71) | 0.6636 | 0.51 (0.11 to 2.39) | 0.3896 | Reference |  | 0.77 (0.56 to 1.04) | 0.0925 |
| Educational attainment of neighborhood (% high school or greater) | <= 80% | Reference |  | Reference |  | Reference |  | Reference |  | 1.24 (0.58 to 2.65) | 0.5778 | Reference |  |
|  | > 80% | 1.05 (0.92 to 1.19) | 0.4864 | 0.89 (0.75 to 1.07) | 0.2095 | 1.06 (0.86 to 1.32) | 0.5638 | 0.71 (0.38 to 1.33) | 0.2845 | Reference |  | 1.03 (0.89 to 1.18) | 0.7043 |
| Median neighborhood income | <= 46k | Reference |  | Reference |  | Reference |  | Reference |  | 0.87 (0.4 to 1.89) | 0.7218 | Reference |  |
|  | > 46k | 1.03 (0.91 to 1.17) | 0.6018 | 0.9 (0.75 to 1.08) | 0.2603 | 1.17 (0.94 to 1.45) | 0.1544 | 1.15 (0.61 to 2.17) | 0.6738 |  |  | 0.99 (0.86 to 1.14) | 0.8417 |

**C**

|  |  | **Adenocarcinoma** | | **Squamous cell cancer** | | **Small cell liung cancer** | | **Carcinoid/Large cell neuroendocrine** | | **Large cell carcinoma** | | **Others** | |
| --- | --- | --- | --- | --- | --- | --- | --- | --- | --- | --- | --- | --- | --- |
| **Variable** | **Category** | **Odds Ratio (95% Confidence Limit)** | **P value** | **Odds Ratio (95% Confidence Limit)** | **P value** | **Odds Ratio (95% Confidence Limit)** | **P value** | **Odds Ratio (95% Confidence Limit)** | **P value** | **Odds Ratio (95% Confidence Limit)** | **P value** | **Odds Ratio (95% Confidence Limit)** | **P value** |
| Age group | <=60 | Reference |  | Reference |  | Reference |  | Reference |  | Reference |  | Reference |  |
|  | 61-70 | 0.86 (0.68 to 1.09) | 0.2255 | 0.58 (0.38 to 0.88) | 0.0103 | 0.82 (0.35 to 1.9) | 0.6369 | 0.32 (0.13 to 0.82) | 0.0175 | 0.45 (0.26 to 0.8) | 0.0064 | 0.45 (0.26 to 0.8) | 0.0064 |
|  | 71-80 | 0.43 (0.34 to 0.55) | <.0001 | 0.44 (0.29 to 0.68) | 0.0002 | 0.62 (0.27 to 1.45) | 0.2704 | 0.12 (0.05 to 0.33) | <.0001 | 0.17 (0.09 to 0.32) | <.0001 | 0.17 (0.09 to 0.32) | <.0001 |
|  | >80 | 0.1 (0.07 to 0.15) | <.0001 | 0.15 (0.08 to 0.26) | <.0001 | 0.67 (0.19 to 2.3) | 0.5223 | 0 (0 to 0.04) | <.0001 | 0.04 (0.02 to 0.12) | <.0001 | 0.04 (0.02 to 0.12) | <.0001 |
| Sex | Female | Reference |  | Reference |  | Reference |  | Reference |  | Reference |  | Reference |  |
|  | Male | 0.67 (0.56 to 0.8) | <.0001 | 1.2 (0.91 to 1.58) | 0.1898 | 1.49 (0.78 to 2.84) | 0.2251 | 0.84 (0.39 to 1.81) | 0.6556 | 1.29 (0.82 to 2.02) | 0.2678 | 1.29 (0.82 to 2.02) | 0.2678 |
| Year of diagnosis | Continuous | 1.02 (1 to 1.05) | 0.0771 | 1.03 (0.99 to 1.08) | 0.0936 | 1.17 (1.06 to 1.29) | 0.0016 | 0.87 (0.76 to 0.99) | 0.0327 | 1.06 (0.99 to 1.13) | 0.117 | 1.06 (0.99 to 1.13) | 0.117 |
| CCI score | 0 | Reference |  | Reference |  | Reference |  | Reference |  | Reference |  | Reference |  |
|  | 1 | 0.91 (0.66 to 1.27) | 0.5807 | 0.76 (0.43 to 1.33) | 0.3361 | 1.01 (0.32 to 3.26) | 0.9805 | 3.1 (0.86 to 11.13) | 0.0835 | 0.46 (0.19 to 1.12) | 0.088 | 0.46 (0.19 to 1.12) | 0.088 |
|  | 2 | 0.86 (0.67 to 1.09) | 0.2125 | 0.54 (0.34 to 0.84) | 0.0069 | 0.83 (0.31 to 2.25) | 0.7167 | 2.71 (0.97 to 7.54) | 0.056 | 1.14 (0.59 to 2.18) | 0.697 | 1.14 (0.59 to 2.18) | 0.697 |
| CVD | Without CVD | Reference |  | Reference |  | Reference |  | Reference |  | Reference |  | Reference |  |
|  | With CVD | 0.6 (0.49 to 0.75) | <.0001 | 0.5 (0.37 to 0.68) | <.0001 | 0.47 (0.21 to 1.06) | 0.0703 | 0.78 (0.29 to 2.11) | 0.6222 | 0.45 (0.26 to 0.78) | 0.0046 | 0.45 (0.26 to 0.78) | 0.0046 |
| Stage | I | Reference |  | Reference |  | Reference |  | Reference |  | Reference |  | Reference |  |
|  | II | 0.93 (0.63 to 1.36) | 0.7128 | 0.54 (0.36 to 0.81) | 0.0031 | 0.4 (0.14 to 1.1) | 0.0768 | 1.23 (0.25 to 5.89) | 0.7998 | 1.64 (0.82 to 3.28) | 0.1643 | 1.64 (0.82 to 3.28) | 0.1643 |
|  | III | 0.05 (0.04 to 0.06) | <.0001 | 0.04 (0.03 to 0.05) | <.0001 | 0.03 (0.01 to 0.06) | <.0001 | 0.06 (0.02 to 0.16) | <.0001 | 0.1 (0.05 to 0.19) | <.0001 | 0.1 (0.05 to 0.19) | <.0001 |
|  | IV | 0 (0 to 0) | <.0001 | 0 (0 to 0.01) | <.0001 | 0 (0 to 0.01) | <.0001 | 0 (0 to 0) | <.0001 | 0.01 (0 to 0.02) | <.0001 | 0.01 (0 to 0.02) | <.0001 |
|  | UNK | 0.01 (0.01 to 0.03) | <.0001 | 0.02 (0.01 to 0.06) | <.0001 | 0.2 (0.05 to 0.85) | 0.0287 | 0.15 (0.05 to 0.5) | 0.0019 | 0.39 (0.18 to 0.81) | 0.0123 | 0.39 (0.18 to 0.81) | 0.0123 |
| Chemo | 0 | Reference |  | Reference |  | Reference |  | Reference |  | Reference |  | Reference |  |
|  | 1 | 1.32 (1.06 to 1.64) | 0.0123 | 3.28 (2.26 to 4.76) | <.0001 | 1.71 (0.76 to 3.85) | 0.1976 | 0.8 (0.29 to 2.19) | 0.659 | 4.78 (2.65 to 8.61) | <.0001 | 4.78 (2.65 to 8.61) | <.0001 |
| Radiation | 0 | Reference |  | Reference |  | Reference |  | Reference |  | Reference |  | Reference |  |
|  | 1 | 0.04 (0.03 to 0.05) | <.0001 | 0.02 (0.01 to 0.03) | <.0001 | 0.11 (0.05 to 0.26) | <.0001 | 0.04 (0.01 to 0.17) | <.0001 | 0.14 (0.07 to 0.29) | <.0001 | 0.14 (0.07 to 0.29) | <.0001 |
| Driving time to the nearest cancer center (minutes) | 1 | Reference |  | Reference |  | Reference |  | Reference |  | Reference |  | Reference |  |
|  | 2 | 1.35 (0.96 to 1.88) | 0.0807 | 1.54 (0.95 to 2.51) | 0.0811 | 5.58 (1.52 to 20.55) | 0.0097 | 2.81 (0.71 to 11.16) | 0.1427 | 1.63 (0.67 to 3.97) | 0.2845 | 1.63 (0.67 to 3.97) | 0.2845 |
|  | 3 | 1.21 (0.74 to 1.98) | 0.4499 | 0.98 (0.48 to 2.02) | 0.9664 | 14.44 (2.56 to 81.48) | 0.0025 | 3.3 (0.15 to 74.95) | 0.4535 | 1.06 (0.31 to 3.7) | 0.9225 | 1.06 (0.31 to 3.7) | 0.9225 |
| Zone name | Calgary | Reference |  | Reference |  | Reference |  | Reference |  | Reference |  | Reference |  |
|  | Central | 1.06 (0.77 to 1.47) | 0.7022 | 0.74 (0.46 to 1.21) | 0.2312 | 0.38 (0.1 to 1.4) | 0.1454 | 0.25 (0.08 to 0.8) | 0.0189 | 0.97 (0.38 to 2.42) | 0.9396 | 0.97 (0.38 to 2.42) | 0.9396 |
|  | Edmonton | 1.99 (1.6 to 2.48) | <.0001 | 1.77 (1.25 to 2.5) | 0.0012 | 2.56 (1.06 to 6.18) | 0.0364 | 1.81 (0.69 to 4.7) | 0.2263 | 1.89 (1.04 to 3.42) | 0.0355 | 1.89 (1.04 to 3.42) | 0.0355 |
|  | North | 1.21 (0.72 to 2.01) | 0.4727 | 1.78 (0.83 to 3.81) | 0.1401 | 0.17 (0.03 to 1.1) | 0.0631 | 0.31 (0.01 to 6.96) | 0.4634 | 1.79 (0.5 to 6.42) | 0.3709 | 1.79 (0.5 to 6.42) | 0.3709 |
|  | South | 0.5 (0.33 to 0.76) | 0.0011 | 0.7 (0.4 to 1.23) | 0.2149 | 0.11 (0.01 to 0.95) | 0.0448 | 0.51 (0.09 to 2.94) | 0.4474 | 0.58 (0.14 to 2.43) | 0.4603 | 0.58 (0.14 to 2.43) | 0.4603 |
| Educational attainment of neighborhood (% high school or greater) | <= 80% | Reference |  | Reference |  | Reference |  | Reference |  | Reference |  | Reference |  |
|  | > 80% | 1.11 (0.9 to 1.36) | 0.3288 | 0.99 (0.73 to 1.34) | 0.9427 | 0.83 (0.42 to 1.67) | 0.609 | 0.78 (0.33 to 1.82) | 0.5673 | 1.07 (0.63 to 1.81) | 0.8033 | 1.07 (0.63 to 1.81) | 0.8033 |
| Median neighborhood income | <= 46k | Reference |  | Reference |  | Reference |  | Reference |  | Reference |  | Reference |  |
|  | > 46k | 1.12 (0.91 to 1.36) | 0.2823 | 1.04 (0.77 to 1.4) | 0.8189 | 1.44 (0.72 to 2.88) | 0.302 | 1.39 (0.6 to 3.2) | 0.4449 | 1.62 (0.97 to 2.71) | 0.063 | 1.62 (0.97 to 2.71) | 0.063 |

**Supplemental Table 2: Age stratified logistic regression analysis to predict the likelihood of treatment (A: Chemotherapy, B: Radiotherapy, C: Surgery)**

A

| **Age** |  | **<=60 years** |  | **61-70 years** |  | **71-80 years** |  | **> 80 years** |  |
| --- | --- | --- | --- | --- | --- | --- | --- | --- | --- |
| **Variable** | **Category** | **Odds Ratio (95% Confidence Limit)** | **P value** | **Odds Ratio (95% Confidence Limit)** | **P value** | **Odds Ratio (95% Confidence Limit)** | **P value** | **Odds Ratio (95% Confidence Limit)** | **P value** |
| Sex | Female | Reference |  | Reference |  | Reference |  | Reference |  |
|  | Male | 0.77 (0.67 to 0.88) | 0.0001 | 0.95 (0.84 to 1.07) | 0.3898 | 1.07 (0.93 to 1.23) | 0.3279 | 1.04 (0.75 to 1.45) | 0.8095 |
| Year of diagnosis | Continuous | 1.02 (1 to 1.04) | 0.0441 | 0.99 (0.98 to 1.01) | 0.4546 | 1.04 (1.02 to 1.06) | 0.0004 | 1.05 (0.99 to 1.1) | 0.0829 |
| CCI score | 0 | Reference |  | Reference |  | Reference |  | Reference |  |
|  | 1 | 0.85 (0.66 to 1.1) | 0.2129 | 0.73 (0.57 to 0.92) | 0.0076 | 0.87 (0.66 to 1.15) | 0.3341 | 0.59 (0.3 to 1.18) | 0.1367 |
|  | 2 | 0.72 (0.6 to 0.86) | 0.0003 | 0.67 (0.56 to 0.8) | <.0001 | 0.67 (0.54 to 0.83) | 0.0003 | 0.59 (0.35 to 0.99) | 0.0449 |
| CVD | Without CVD | Reference |  | Reference |  | Reference |  | Reference |  |
|  | With CVD | 0.41 (0.33 to 0.5) | <.0001 | 0.56 (0.48 to 0.65) | <.0001 | 0.5 (0.43 to 0.59) | <.0001 | 0.73 (0.52 to 1.03) | 0.0694 |
| Histology | Adenocarcinoma | Reference |  | Reference |  | Reference |  | Reference |  |
|  | Carcinoid/LCNE | 0.58 (0.4 to 0.85) | 0.0054 | 1.14 (0.78 to 1.66) | 0.5135 | 1.49 (0.95 to 2.36) | 0.0836 | 1.86 (0.73 to 4.69) | 0.1912 |
|  | Large cell ca | 0.81 (0.49 to 1.33) | 0.4032 | 1.05 (0.62 to 1.77) | 0.8511 | 0.83 (0.42 to 1.64) | 0.595 | 0 (0 to .) | 0.9831 |
|  | Others | 0.59 (0.49 to 0.71) | <.0001 | 0.51 (0.43 to 0.61) | <.0001 | 0.36 (0.29 to 0.44) | <.0001 | 0.14 (0.08 to 0.25) | <.0001 |
|  | SCC | 0.7 (0.57 to 0.87) | 0.0011 | 0.7 (0.59 to 0.83) | <.0001 | 0.59 (0.48 to 0.72) | <.0001 | 0.23 (0.11 to 0.47) | <.0001 |
|  | SCLC | 5.71 (4.54 to 7.18) | <.0001 | 6.6 (5.44 to 7.99) | <.0001 | 7.31 (6.03 to 8.85) | <.0001 | 8.18 (5.54 to 12.07) | <.0001 |
| Stage | I | Reference |  | Reference |  | Reference |  | Reference |  |
|  | II | 10.07 (6.91 to 14.68) | <.0001 | 9.47 (6.9 to 13.01) | <.0001 | 7.97 (5.47 to 11.61) | <.0001 | 1.76 (0.34 to 9.09) | 0.4987 |
|  | III | 9.49 (6.88 to 13.08) | <.0001 | 9.58 (7.29 to 12.6) | <.0001 | 9.5 (6.91 to 13.06) | <.0001 | 3.62 (1.64 to 7.97) | 0.0014 |
|  | IV | 3.89 (2.79 to 5.41) | <.0001 | 4.98 (3.77 to 6.58) | <.0001 | 6.26 (4.52 to 8.65) | <.0001 | 3.4 (1.56 to 7.42) | 0.0021 |
|  | UNK | 1.31 (0.7 to 2.45) | 0.4034 | 1.97 (1.07 to 3.66) | 0.0307 | 1.47 (0.68 to 3.19) | 0.3242 | 0.89 (0.17 to 4.59) | 0.8935 |
| Surgery type | no surgery | Reference |  | Reference |  | Reference |  | Reference |  |
|  | had surgery | 0.96 (0.72 to 1.3) | 0.8043 | 1.44 (1.12 to 1.85) | 0.0041 | 1.55 (1.16 to 2.07) | 0.0028 | 0.31 (0.07 to 1.39) | 0.1245 |
| Radiation | 0 | Reference |  | Reference |  | Reference |  | Reference |  |
|  | 1 | 1.1 (0.94 to 1.28) | 0.2203 | 1.13 (0.99 to 1.3) | 0.0725 | 1.42 (1.21 to 1.67) | <.0001 | 1.2 (0.78 to 1.82) | 0.4064 |
| Surgery institution type | academic | Reference |  | Reference |  | Reference |  | Reference |  |
|  | community | 0.75 (0.63 to 0.88) | 0.0005 | 0.76 (0.65 to 0.88) | 0.0002 | 0.84 (0.71 to 1) | 0.0506 | 1.12 (0.75 to 1.66) | 0.5862 |
| Driving time to the nearest cancer center (minutes) | 1 | Reference |  | Reference |  | Reference |  | Reference |  |
|  | 2 | 0.79 (0.61 to 1.02) | 0.0723 | 0.96 (0.77 to 1.19) | 0.7084 | 0.93 (0.73 to 1.2) | 0.5864 | 0.84 (0.45 to 1.55) | 0.5793 |
|  | 3 | 0.77 (0.52 to 1.15) | 0.2008 | 0.97 (0.71 to 1.34) | 0.8717 | 0.89 (0.61 to 1.29) | 0.5311 | 1.11 (0.44 to 2.78) | 0.8257 |
| Zone name | Calgary | Reference |  | Reference |  | Reference |  | Reference |  |
|  | Central | 1.47 (1.15 to 1.9) | 0.0026 | 1.33 (1.06 to 1.67) | 0.0129 | 1.43 (1.11 to 1.86) | 0.006 | 0.98 (0.53 to 1.79) | 0.938 |
|  | Edmonton | 1.01 (0.85 to 1.19) | 0.9452 | 1.06 (0.91 to 1.23) | 0.4893 | 0.97 (0.81 to 1.15) | 0.7078 | 0.5 (0.33 to 0.77) | 0.0015 |
|  | North | 1.43 (0.95 to 2.17) | 0.0896 | 1.15 (0.82 to 1.61) | 0.4054 | 1.03 (0.69 to 1.54) | 0.8857 | 0.92 (0.34 to 2.48) | 0.8706 |
|  | South | 1.43 (1.05 to 1.96) | 0.0244 | 1.26 (0.97 to 1.65) | 0.0875 | 1.15 (0.85 to 1.57) | 0.3634 | 0.86 (0.43 to 1.71) | 0.667 |
| Educational attainment of neighborhood (% high school or greater) | <= 80% | Reference |  | Reference |  | Reference |  | Reference |  |
|  | > 80% | 1.05 (0.9 to 1.22) | 0.5384 | 1.05 (0.92 to 1.21) | 0.4415 | 1.08 (0.92 to 1.26) | 0.3756 | 1.01 (0.69 to 1.48) | 0.9664 |
| Median neighborhood income | <= 46k | Reference |  | Reference |  | Reference |  | Reference |  |
|  | > 46k | 1.29 (1.1 to 1.5) | 0.0012 | 1.29 (1.13 to 1.48) | 0.0002 | 1.04 (0.88 to 1.22) | 0.6627 | 1.03 (0.71 to 1.52) | 0.8629 |

B

| **Age** |  | **<= 60 years** |  | **61-70 years** |  | **71-80 years** |  | **> 80 years** |  |
| --- | --- | --- | --- | --- | --- | --- | --- | --- | --- |
| **Variable** | **Category** | **Odds Ratio (95% Confidence Limit)** | **P value** | **Odds Ratio (95% Confidence Limit)** | **P value** | **Odds Ratio (95% Confidence Limit)** | **P value** | **Odds Ratio (95% Confidence Limit)** | **P value** |
| Sex | Female | Reference |  | Reference |  | Reference |  | Reference |  |
|  | Male | 0.97 (0.84 to 1.12) | 0.6969 | 1.21 (1.08 to 1.37) | 0.0017 | 1.16 (1.03 to 1.31) | 0.012 | 1.12 (0.93 to 1.35) | 0.2158 |
| Year of diagnosis | Continuous | 0.98 (0.96 to 1) | 0.0675 | 1 (0.99 to 1.02) | 0.7784 | 1 (0.98 to 1.02) | 0.8596 | 1 (0.98 to 1.03) | 0.851 |
| CCI score | 0 | Reference |  | Reference |  | Reference |  | Reference |  |
|  | 1 | 0.85 (0.64 to 1.13) | 0.2743 | 1.38 (1.08 to 1.76) | 0.0107 | 0.95 (0.73 to 1.22) | 0.6682 | 0.8 (0.56 to 1.16) | 0.245 |
|  | 2 | 1.26 (1.03 to 1.53) | 0.0213 | 1.34 (1.11 to 1.62) | 0.0026 | 1.12 (0.92 to 1.38) | 0.2643 | 0.85 (0.63 to 1.15) | 0.2899 |
| CVD | Without CVD | Reference |  | Reference |  | Reference |  | Reference |  |
|  | With CVD | 0.66 (0.54 to 0.8) | <.0001 | 0.75 (0.65 to 0.86) | <.0001 | 0.83 (0.74 to 0.94) | 0.0035 | 0.62 (0.51 to 0.74) | <.0001 |
| Histology | Adenocarcinoma | Reference |  | Reference |  | Reference |  | Reference |  |
|  | Carcinoid/LCNE | 1.01 (0.63 to 1.62) | 0.9574 | 0.62 (0.37 to 1.02) | 0.0615 | 0.7 (0.41 to 1.2) | 0.1962 | 0.83 (0.37 to 1.88) | 0.6537 |
|  | Large cell ca | 1.5 (0.86 to 2.62) | 0.1578 | 2.07 (1.15 to 3.73) | 0.0152 | 2.6 (1.48 to 4.57) | 0.0008 | 1.75 (0.76 to 4.03) | 0.1858 |
|  | Others | 1.37 (1.14 to 1.64) | 0.0009 | 0.84 (0.72 to 0.98) | 0.0311 | 0.67 (0.58 to 0.78) | <.0001 | 0.39 (0.31 to 0.49) | <.0001 |
|  | SCC | 2.54 (2.03 to 3.19) | <.0001 | 1.83 (1.53 to 2.17) | <.0001 | 2.33 (1.98 to 2.74) | <.0001 | 2.79 (2.16 to 3.61) | <.0001 |
|  | SCLC | 0.86 (0.7 to 1.06) | 0.1614 | 0.78 (0.65 to 0.94) | 0.0102 | 0.59 (0.48 to 0.73) | <.0001 | 0.54 (0.35 to 0.82) | 0.0037 |
| Stage | I | Reference |  | Reference |  | Reference |  | Reference |  |
|  | II | 2.83 (1.64 to 4.9) | 0.0002 | 1.8 (1.17 to 2.76) | 0.0069 | 1.25 (0.88 to 1.77) | 0.2119 | 1.4 (0.84 to 2.34) | 0.2002 |
|  | III | 5.28 (3.51 to 7.95) | <.0001 | 2.06 (1.58 to 2.67) | <.0001 | 0.98 (0.8 to 1.19) | 0.8176 | 0.58 (0.45 to 0.75) | <.0001 |
|  | IV | 1.29 (0.86 to 1.93) | 0.2181 | 0.63 (0.49 to 0.81) | 0.0004 | 0.39 (0.32 to 0.47) | <.0001 | 0.28 (0.22 to 0.37) | <.0001 |
|  | UNK | 0.79 (0.37 to 1.68) | 0.5348 | 0.35 (0.19 to 0.63) | 0.0005 | 0.17 (0.11 to 0.28) | <.0001 | 0.17 (0.09 to 0.3) | <.0001 |
| Surgery type | no surgery | Reference |  | Reference |  | Reference |  | Reference |  |
|  | had surgery | 0.06 (0.04 to 0.09) | <.0001 | 0.03 (0.02 to 0.04) | <.0001 | 0.01 (0.01 to 0.02) | <.0001 | 0.01 (0 to 0.03) | <.0001 |
| Chemo | 0 | Reference |  | Reference |  | Reference |  | Reference |  |
|  | 1 | 1.12 (0.96 to 1.3) | 0.1647 | 1.17 (1.02 to 1.34) | 0.024 | 1.43 (1.22 to 1.68) | <.0001 | 1.12 (0.74 to 1.69) | 0.5953 |
| Surgery institution type | academic | Reference |  | Reference |  | Reference |  | Reference |  |
|  | community | 0.75 (0.64 to 0.89) | 0.0007 | 1.03 (0.9 to 1.19) | 0.6369 | 0.98 (0.86 to 1.13) | 0.8189 | 0.77 (0.62 to 0.95) | 0.0166 |
| Driving time to the nearest cancer center (minutes) | 1 | Reference |  | Reference |  | Reference |  | Reference |  |
|  | 2 | 0.98 (0.75 to 1.29) | 0.8891 | 1.01 (0.81 to 1.24) | 0.9589 | 0.97 (0.78 to 1.19) | 0.7443 | 0.97 (0.69 to 1.38) | 0.8789 |
|  | 3 | 1.26 (0.83 to 1.9) | 0.2766 | 1.03 (0.75 to 1.4) | 0.8693 | 0.77 (0.55 to 1.08) | 0.1265 | 0.43 (0.23 to 0.81) | 0.0096 |
| Zone name | Calgary | Reference |  | Reference |  | Reference |  | Reference |  |
|  | Central | 0.88 (0.67 to 1.15) | 0.3406 | 0.69 (0.55 to 0.86) | 0.0014 | 0.68 (0.55 to 0.85) | 0.0007 | 1.08 (0.76 to 1.55) | 0.6639 |
|  | Edmonton | 1.21 (1.02 to 1.44) | 0.0308 | 0.94 (0.8 to 1.09) | 0.4094 | 0.99 (0.85 to 1.15) | 0.8846 | 1.34 (1.08 to 1.67) | 0.0085 |
|  | North | 0.9 (0.58 to 1.39) | 0.6335 | 0.85 (0.61 to 1.18) | 0.3193 | 0.94 (0.67 to 1.34) | 0.748 | 1.98 (1.05 to 3.74) | 0.0357 |
|  | South | 1.09 (0.79 to 1.51) | 0.5988 | 0.94 (0.72 to 1.22) | 0.6171 | 0.8 (0.62 to 1.04) | 0.0922 | 0.86 (0.57 to 1.3) | 0.4799 |
| Educational attainment of neighborhood (% high school or greater) | <= 80% | Reference |  | Reference |  | Reference |  | Reference |  |
|  | > 80% | 0.97 (0.83 to 1.14) | 0.7163 | 0.91 (0.79 to 1.04) | 0.1509 | 1.04 (0.91 to 1.2) | 0.5287 | 1.23 (0.99 to 1.52) | 0.062 |
| Median neighborhood income | <= 46k | Reference |  | Reference |  | Reference |  | Reference |  |
|  | > 46k | 0.97 (0.83 to 1.14) | 0.6988 | 1.15 (1 to 1.31) | 0.0495 | 0.88 (0.76 to 1) | 0.0568 | 1.08 (0.87 to 1.33) | 0.4931 |

C

| **Age** |  | **<=60 years** |  | **61-70 years** |  | **71-80 years** |  | **> 80 years** |  |
| --- | --- | --- | --- | --- | --- | --- | --- | --- | --- |
| **Variable** | **Category** | **Odds Ratio (95% Confidence Limit)** | **P value** | **Odds Ratio (95% Confidence Limit)** | **P value** | **Odds Ratio (95% Confidence Limit)** | **P value** | **Odds Ratio (95% Confidence Limit)** | **P value** |
| Sex | Female | Reference |  | Reference |  | Reference |  | Reference |  |
|  | Male | 0.67 (0.47 to 0.94) | 0.0215 | 0.77 (0.58 to 1.01) | 0.0592 | 1.09 (0.83 to 1.45) | 0.533 | 0.98 (0.56 to 1.7) | 0.9449 |
| Year of diagnosis | Continuous | 1.02 (0.97 to 1.07) | 0.4946 | 1.02 (0.98 to 1.06) | 0.345 | 1.01 (0.97 to 1.06) | 0.492 | 1.02 (0.94 to 1.12) | 0.5841 |
| CCI score | 0 | Reference |  | Reference |  | Reference |  | Reference |  |
|  | 1 | 0.73 (0.39 to 1.34) | 0.3025 | 0.91 (0.54 to 1.53) | 0.7241 | 1.08 (0.62 to 1.86) | 0.7899 | 0.35 (0.11 to 1.11) | 0.0738 |
|  | 2 | 0.73 (0.47 to 1.13) | 0.1568 | 0.84 (0.56 to 1.26) | 0.3902 | 0.85 (0.55 to 1.32) | 0.4647 | 0.7 (0.29 to 1.7) | 0.4308 |
| CVD | Without CVD | Reference |  | Reference |  | Reference |  | Reference |  |
|  | With CVD | 0.46 (0.28 to 0.75) | 0.0021 | 0.63 (0.46 to 0.87) | 0.0052 | 0.58 (0.43 to 0.78) | 0.0004 | 0.36 (0.2 to 0.63) | 0.0004 |
| Histology | Adenocarcinoma | Reference |  | Reference |  | Reference |  | Reference |  |
|  | Carcinoid/LCNE | 2.6 (1.28 to 5.29) | 0.0083 | 1.25 (0.59 to 2.64) | 0.5555 | 2.01 (0.87 to 4.6) | 0.1004 | 0.35 (0 to 40.13) | 0.6647 |
|  | Large cell ca | 1.37 (0.41 to 4.6) | 0.6095 | 6.54 (2.32 to 18.49) | 0.0004 | 2.3 (0.45 to 11.88) | 0.3184 | 21.64 (2.66 to 176.14) | 0.0041 |
|  | Others | 0.09 (0.05 to 0.16) | <.0001 | 0.04 (0.03 to 0.07) | <.0001 | 0.02 (0.01 to 0.03) | <.0001 | 0.02 (0.01 to 0.04) | <.0001 |
|  | SCC | 0.66 (0.4 to 1.07) | 0.0922 | 0.61 (0.43 to 0.86) | 0.0052 | 0.67 (0.48 to 0.94) | 0.0193 | 1.05 (0.54 to 2.04) | 0.8773 |
|  | SCLC | 0.06 (0.03 to 0.12) | <.0001 | 0.07 (0.03 to 0.13) | <.0001 | 0.1 (0.05 to 0.18) | <.0001 | 0.62 (0.19 to 2.05) | 0.4314 |
| Stage | I | Reference |  | Reference |  | Reference |  | Reference |  |
|  | II | 0.75 (0.36 to 1.57) | 0.4492 | 1.23 (0.71 to 2.14) | 0.4534 | 0.85 (0.5 to 1.44) | 0.5502 | 0.76 (0.29 to 2.01) | 0.5756 |
|  | III | 0.05 (0.03 to 0.09) | <.0001 | 0.07 (0.05 to 0.1) | <.0001 | 0.07 (0.05 to 0.1) | <.0001 | 0.06 (0.03 to 0.12) | <.0001 |
|  | IV | 0 (0 to 0) | <.0001 | 0.01 (0 to 0.01) | <.0001 | 0 (0 to 0.01) | <.0001 | 0 (0 to 0.01) | <.0001 |
|  | UNK | 0.17 (0.07 to 0.4) | <.0001 | 0.14 (0.06 to 0.33) | <.0001 | 0.05 (0.02 to 0.13) | <.0001 | 0.08 (0.02 to 0.3) | 0.0003 |
| Chemo | 0 | Reference |  | Reference |  | Reference |  | Reference |  |
|  | 1 | 1.52 (1.03 to 2.24) | 0.0328 | 1.6 (1.17 to 2.18) | 0.0032 | 1.92 (1.32 to 2.81) | 0.0007 | 0.48 (0.08 to 2.84) | 0.4186 |
| Radiation | 0 | Reference |  | Reference |  | Reference |  | Reference |  |
|  | 1 | 0.09 (0.06 to 0.14) | <.0001 | 0.04 (0.03 to 0.06) | <.0001 | 0.02 (0.01 to 0.03) | <.0001 | 0.01 (0 to 0.04) | <.0001 |
| Surgery institution type | academic | Reference |  | Reference |  | Reference |  | Reference |  |
|  | community | 0.01 (0 to 0.01) | <.0001 | 0 (0 to 0.01) | <.0001 | 0 (0 to 0) | <.0001 | 0 (0 to 0.01) | <.0001 |
| Driving time to the nearest cancer center (minutes) | 1 | Reference |  | Reference |  | Reference |  | Reference |  |
|  | 2 | 1.62 (0.71 to 3.7) | 0.2529 | 1.52 (0.77 to 2.96) | 0.2245 | 1.25 (0.58 to 2.7) | 0.5732 | 1.92 (0.21 to 17.5) | 0.564 |
|  | 3 | 1.01 (0.31 to 3.31) | 0.992 | 0.66 (0.27 to 1.64) | 0.3745 | 0.98 (0.37 to 2.58) | 0.9595 | 1.46 (0.08 to 25.89) | 0.797 |
| Zone name | CALGARY | Reference |  | Reference |  | Reference |  | Reference |  |
|  | CENTRAL | 4.17 (1.9 to 9.15) | 0.0004 | 3.87 (2.07 to 7.25) | <.0001 | 11.41 (5.43 to 24.01) | <.0001 | 19.63 (3.04 to 126.66) | 0.0018 |
|  | EDMONTON | 1.57 (1.08 to 2.29) | 0.0194 | 1.66 (1.22 to 2.27) | 0.0014 | 2.15 (1.57 to 2.94) | <.0001 | 4.64 (2.44 to 8.82) | <.0001 |
|  | NORTH | 6.24 (1.85 to 21.01) | 0.0031 | 5.83 (2.29 to 14.8) | 0.0002 | 9.95 (3.68 to 26.9) | <.0001 | 27.02 (1.65 to 442.22) | 0.0208 |
|  | SOUTH | 4.38 (1.5 to 12.8) | 0.0069 | 3.3 (1.38 to 7.93) | 0.0075 | 4.19 (1.54 to 11.37) | 0.0049 | 33.76 (1.94 to 586.21) | 0.0157 |
| Educational attainment of neighborhood (% high school or greater) | <= 80% | Reference |  | Reference |  | Reference |  | Reference |  |
|  | > 80% | 0.92 (0.63 to 1.35) | 0.6734 | 0.93 (0.68 to 1.26) | 0.6257 | 1.08 (0.78 to 1.5) | 0.6375 | 0.85 (0.44 to 1.61) | 0.612 |
| Median neighborhood income | <= 46k | Reference |  | Reference |  | Reference |  | Reference |  |
|  | > 46k | 1.07 (0.73 to 1.56) | 0.7302 | 1.59 (1.18 to 2.15) | 0.0026 | 0.99 (0.72 to 1.35) | 0.9432 | 1.08 (0.58 to 2.01) | 0.8046 |
